# RNA-Seq Analysis Using *De Novo* Transcriptome Assembly as a Reference for the Salmon Louse *Caligus rogercresseyi*

**DOI:** 10.1371/journal.pone.0092239

**Published:** 2014-04-01

**Authors:** Cristian Gallardo-Escárate, Valentina Valenzuela-Muñoz, Gustavo Nuñez-Acuña

**Affiliations:** Laboratory of Biotechnology and Aquatic Genomics, Interdisciplinary Center for Aquaculture Research (INCAR), University of Concepción, Concepción, Chile; University of North Carolina at Charlotte, United States of America

## Abstract

Despite the economic and environmental impacts that sea lice infestations have on salmon farming worldwide, genomic data generated by high-throughput transcriptome sequencing for different developmental stages, sexes, and strains of sea lice is still limited or unknown. In this study, RNA-seq analysis was performed using *de novo* transcriptome assembly as a reference for evidenced transcriptional changes from six developmental stages of the salmon louse *Caligus rogercresseyi*. EST-datasets were generated from the nauplius I, nauplius II, copepodid and chalimus stages and from female and male adults using MiSeq Illumina sequencing. A total of 151,788,682 transcripts were yielded, which were assembled into 83,444 high quality contigs and subsequently annotated into roughly 24,000 genes based on known proteins. To identify differential transcription patterns among salmon louse stages, cluster analyses were performed using normalized gene expression values. Herein, four clusters were differentially expressed between nauplius I–II and copepodid stages (604 transcripts), five clusters between copepodid and chalimus stages (2,426 transcripts), and six clusters between female and male adults (2,478 transcripts). Gene ontology analysis revealed that the nauplius I–II, copepodid and chalimus stages are mainly annotated to aminoacid transfer/repair/breakdown, metabolism, molting cycle, and nervous system development. Additionally, genes showing differential transcription in female and male adults were highly related to cytoskeletal and contractile elements, reproduction, cell development, morphogenesis, and transcription-translation processes. The data presented in this study provides the most comprehensive transcriptome resource available for *C. rogercresseyi*, which should be used for future genomic studies linked to host-parasite interactions.

## Introduction

Major challenges facing transcriptomic research in non-model organisms are increasing the speed and accuracy of discovering new genes and metabolic pathways, as well as determining how gene transcription variations are regulated by specific DNA polymorphisms. Understanding the transcriptome is essential for interpreting the functional elements of the genome, for revealing the molecular constituents of cells and tissues, and for understanding complex biological processes such as growth, reproduction, and immune response [1]. Next-generation sequencing (NGS) technologies offer the opportunity to generate genome-wide sequence data sets for a reasonable cost and time [2]–[4]. Although these powerful and rapidly evolving technologies have only been available for a few years, they are already making substantial contributions to the understanding of genome expression and regulation under different conditions. A popular application of NGS is species transcriptome generation, which affords direct access to the coding sequences of many genes and information on their relative expression levels [5]–[7]. NGS transcriptome data analysis is therefore a useful source for mining molecular markers such as SNPs and EST-SSRs [8]–[11].

Salmon lice are naturally occurring parasites for seawater salmon, and, compared to natural conditions, parasite infection and transmission are exacerbated under intensive fish farming. The salmon louse *Caligus rogercresseyi* is the main copepod ectoparasite responsible for significant economic losses of the farmed salmon industry in Chile [12]. This parasite is known to cause surface damage to fish, which results in mucus breakdown and in turn leads to open sores and lesions. A further problem may arise if fish become stressed due to the presence of sea lice [13], [14]. It has been observed that chronic stress in fish may result in immunosuppression and a subsequent increased susceptibility to secondary infections [15]. Moreover, salmon lice infestations have been managed by antiparasite agents including organophosphates [16], [17], pyrethroids [18], hydrogen peroxide [19], and avermectins [20], [21]. However, overexposure to these chemical agents tends to promote drug resistance in wild populations of parasites [22].

These concerns, added to the scarce genomic knowledge of the molecular pathways affected by salmon lice treatments in *C. rogercresseyi*, provide incentive for the scientific community to increase sequencing efforts in order to identify novel candidate genes that could be related to drug resistance and susceptibility. So far, EST datasets have been reported for a few copepod ectoparasites [23]. As of October 2013, the NCBI EST-database retrieved 191,020 EST entries for parasitic copepods; a result that was comprised of 129,250 ESTs for *Lepeophtheirus salmonis*, 32,037 ESTs for *Caligus rogercresseyi*, 14,927 ESTs for *Lernaeocera branchialis*, and 14,806 ESTs for *Caligus clemensi*. These entries have provided a substantial number of sequences that are similar to already reported genes, but a large proportion do not show EST hits with known proteins. Whole transcriptome shotgun sequencing, or RNA sequencing (RNA-seq), tools allow for expression analysis in organisms without previously sequenced genomes, such as marine invertebrates where the majority of species do not have reference genomes available.

In this study, RNA-seq analysis was performed using *de novo* transcriptome assembly as a reference for the salmon louse *C. rogercresseyi*. The goal of this study was to produce whole transcriptome sequences, which would provide pivotal genomic knowledge on the processes involved in the life cycle of the salmon louse. In total, 83,444 transcripts were identified in association with all major signaling pathways and developmental processes of *C. rogercresseyi*. RNA-seq cluster analysis using the MiSeq Illumina platform between larval stages (nauplius I, nauplius II, copepodid and chalimus) and adult individuals (female and male) evidenced a wide diversity of candidate genes related to ontogenetic development, immune response, stress, drug resistance, the nervous system, and reproduction.

## Materials and Methods

### Salmon lice culturing

Female specimens of *C. rogercresseyi* were collected from recently harvested fish at a salmon farm located in Puerto Montt, located in the south of Chile. Individuals were transported back to the laboratory on ice, and their egg strings were then removed and placed in culture buckets supplied with seawater flow at 12°C and with gentle aeration. Eggs were allowed to hatch and develop until the infectious copepodid stage. These were then used to inoculate a tank containing host fish according Bravo [24]. Prior to the collection of salmon lice, fish were anaesthetized. Salmon lice were then harvested for RNA extraction and cDNA library construction. All laboratory infections and culture procedure were carried out under guidelines approved by the ethics committee of University of Concepción and appropriate veterinary supervision.

### Illumina sequencing

The life cycle of *C. rogercresseyi* comprises eight development stages: nauplius 1–2, copepodid, chalimus 1–4 and adult [25]. Herein, twenty individuals from each instars of *C. rogercresseyi* were separately collected. In the case of the chalimus stage, samples from the instars 3–4 were collected. Immediately after sampling, each salmon lice stage were pooled into two biological replicates in 1 mL of RNAlater stabilization solution (Ambion®, USA) and stored at −80°C. Total RNA was extracted from pools using the Ribopure™ kit (Ambion®, Life Technologies™, USA) following the manufacturer's instructions. The concentration and purity were measured with a spectrophotometer (ND-1000, Nanodrop Technologies), and the integrity was visualized with electrophoresis in MOPS/formaldehyde agarose gels at 1.2% staining with ethidium bromide at 0.001%. RNA was also checked for quality on the Bioanalyzer TapeStation 2200 (Agilent Technologies Inc., USA) using the R6K reagent kit according to the manufacturer's instructions. RNA extracts that presented 260/280 and 260/230 purity indices equal to or greater than 2.0 and integral RNA in electrophoresis and Bioanalyzer measurements (RIN>8) were selected. Subsequently, mRNA pools were precipitated overnight with 2× volume of absolute ethanol and 0.1× volume of 0.3 M sodium acetate at −80°C for cDNA library construction. Following this, double-stranded cDNA libraries were constructed using the TruSeq RNA Sample Preparation kit v2 (Illumina®, USA). Two biological replicates for each developmental stage were separately sequenced by the MiSeq (Illumina®) platform using sequenced runs of 2×250 paired-end reads at the Laboratory of Biotechnology and Aquatic Genomics, Interdisciplinary Center for Aquaculture Research (INCAR), University of Concepción, Chile.

### Data deposition

The cleaned short read sequences were deposited in the Sequence Read Archive (SRA) (http://www.ncbi.nlm.nih.gov/sra) under the accession number SRR1106551. The *de novo* assembly sequence data is available from corresponding author on request.

### *De novo* transcriptome assembly

The raw data for each pool of samples were separately trimmed and *de novo* assembled in a unique file using the CLC Genomics Workbench software (Version 6.0.1, CLC Bio, Denmark). The overlap settings for this assembly were a mismatch cost of 2, an insert cost of 3, a minimum contig length of 200 base pairs (bp), a similarity of 0.8, and a trimming quality score of 0.05. This assembly yielded 83,444 contigs that were annotated according to Gene Ontology terms with the Blast2Go program [26], that was executed as a plugin of CLC by mapping against the UniprotKB/Swiss-Prot database (http://uniprot.org) with a cutoff E-value of 1E-05. Furthermore, to determine putative gene descriptions, homology searches were carried out through querying the NCBI EST-database using the tBLASTx algorithm. Finally, the assembled sequences were compared to the Kyoto Encyclopedia of Genes and Genomes (KEGG) database [27]. KEGG pathways were assigned to the assembled sequences using the KEGG Automatic Annotation Server (KAAS). The bidirectional best hit (BBH) method was used to obtain KEGG Orthology assignments for each developmental stage of the salmon louse.

### Differential gene expression analysis and clustering

The consensus contigs generated by *de novo* assembly in the previous step were used as a reference for RNA-seq expression analysis. Using the CLC Genomic Workbench software, the readings for each biological replicate were separately mapped against 83,444 contigs. The RNA-seq settings were a minimum length fraction of 0.6 and a minimum similarity fraction (long reads) of 0.5. Then the number of reads per kilobase per million mapped reads (RPKM) was obtained with the same software [28]. This normalized the number of reads to the size of assembled contigs and allowed for assessing the transcripts that were overexpressed among different groups. In order to identify differences between developmental stages, RNA-seq analyses were performed for nauplius I, nauplius II, copepodid and chalimus, and female and male adult stages. Following this, the transcripts that were differentially expressed in comparison to normalized expression values were visualized in a clustering heat map and selected according to the identified cluster. For an optimal comparison of the results, k-means clustering was performed to identify candidate genes involved in specific gene expression patterns. The distance metric was calculated with the Manhattan method, where the mean expression level in 5–6 rounds of k-means clustering was subtracted. Finally, a Volcano plot and Kal's statistical analysis test were used to compare gene expression levels for larval stages and adults in terms of the log_2_ fold change (P<0.0005, FDR corrected).

### Validation by qRT-PCR

Nine genes were chosen for the confirmation of differentially expressed genes by qRT-PCR in the six studied developmental stages. Herein, specific primers were designed from acetoacetyl-CoA synthetase, flotillin, allatostatin precursor protein, tropomyosin, putative cuticle protein, vitellogenin 1, vitellogenin 2, argonaute 1 isoform C and vasa gene (Table S1). The qPCR runs were performed with StepOnePlus™ (Applied Biosystems, Life Technologies, USA) using the comparative ΔCt method. Each reaction was conducted with a volume of 10 µL using the Maxima® SYBR Green/ROX qPCR Master Mix (Thermo Scientific, USA). The amplification conditions were as follows: 95°C for 10 min, 40 cycles at 95°C for 30 s, 60°C for 30 s, and 72°C for 30 s. Three putative housekeeping genes (HKG), *Elongation factor 1-alpha*, *β-actin* and *β-tubulin* were statistically analyzed by NormFinder algorithm to assess their transcriptional expression stability. Here, *β-tubulin* was selected as HKG for gene normalization.

## Results

### Sequencing analysis and assembly from *C. rogercresseyi* transcriptome

Six types of cDNA samples, which represented different developmental stages and adult tissues of *C. rogercresseyi*, were prepared and sequenced using the MiSeq Illumina platform. The sequencing runs yielded a total of 154.84 M reads with an average length of 171 bp. The CLC Genomic Workbench software was used with default parameters to screen for adapter sequences and eliminate poor quality reads. After quality trimming and removal of adapter sequences, 151.78 M reads, representing 97% of the raw reads, remained in the dataset. Of these, 132.5 M reads (88%) wholly or partially assembled into contigs, and 19.26 M reads remained singletons. The remaining reads were excluded from further analyses. The high-throughput sequencing performed for each developmental stage showed similar numbers of yielded reads and average length. Interestingly, the number of singletons did not show major differences among larval and adults stages. The number of nucleotides generated from the *C. rogercresseyi* transcriptome using Illumina technology was up to 25.9 Gigabases (Table 1). *De novo* assembly yielded 83,444 contigs with an average length of 819 bp, of which 58,320 contigs had a length between 300 and 2,000 bp and 25,124 contigs were longer than 2,000 bp. The average coverage among the contigs was 351.1 reads/bp, suggesting that every base pair in the salmon louse transcriptome was sequenced up to 300 times on average. The contigs yielded from the *de novo* assembly performed for each developmental stage ranged from 29,887 in nauplius I to 50,174 in copepodids, with an average length of 823 bp (Table 1). The sequencing results evidenced lower variation between the biological replicates for each stage. For instance, the average coverage did not show significant differences among replicates (data not shown).

**Table 1:** Summary of illumina sequencing from salmon lice *Caligus rogercresseyi* transcriptome.

	Nauplius I	Nauplius II	Copepodid	Chalimus	Female	Male	*De novo* assembly
**ESTs**							
Reads (M)	15.80	17.23	27.17	29.09	30.19	32.29	151.78
Average length (bp)	187	193	190	188	148	142	171
Matched (M)	11.1(71%)	12.6(74%)	23.4(86%)	25.0(86%)	25.9(85%)	27.6(86%)	132.5(88%)
Contigs	29,887	31,778	50,174	42,621	32,172	38,177	83,444
Average length (bp)	978	1,001	707	726	799	729	819
Singletons (M)	4.67	4.59	3.79	4.06	4.30	4.54	19.26
Average length (bp)	126	129	199	197	145	139	149
Number nucleotides (Gb)	2.96	3.34	5.17	5.46	4.47	4.59	25.9

*Two sequencing runs were performed for each sample (biological replicates).

### Transcriptome annotation from *C. rogercresseyi*

Using the BLASTx program, sequence similarity searches of the SwissProt and NR Protein databases showed that 23,841 contigs (28.6% of total contigs) had significant blast matches with E-values≤1e^−5^, making them an annotatable gene set (Table S2). The most abundant BLAST hits were associated with arthropod species such as *Daphia pulex* (13.4%), *Lepeophtheirus salmonis* (7.9%), *Caligus rogercresseyi* (1.9%), *Caligus clemens* (1.6%), *Litopenaeus vannamei* (0.7%), and other crustaceans (10%) like *Calanus finmarchicus*, *Artemia franciscana*, and *Penaeus monodon*, among others. However, the highest hits for species distribution were associated with unknown species (64.4%).

**Figure 1:**
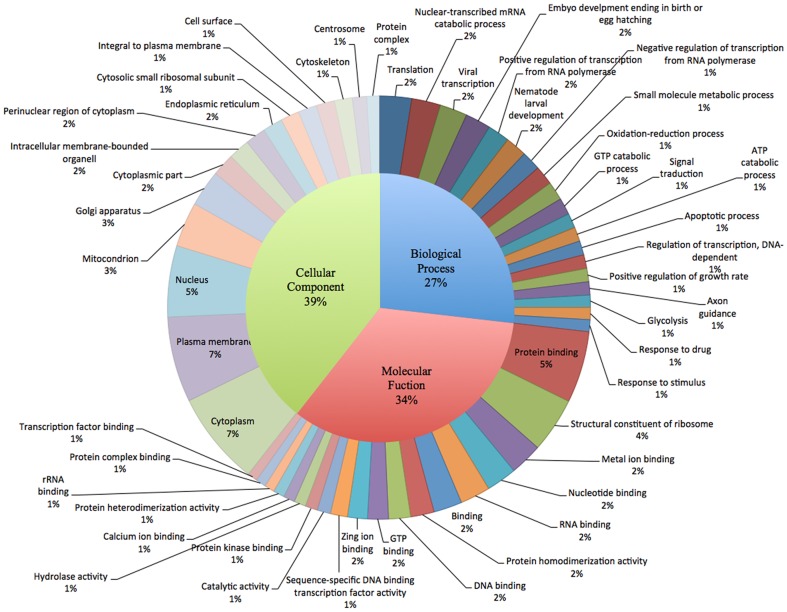
Gene Ontology terms distribution of BLAST hits from the *C. rogercresseyi* transcriptome. Selected GO categories are shown with the top-level division of biological process, molecular function, and molecular function.

Gene Ontology analysis was carried out to explore and summarize the functional categories of the genes sequenced in this study. Among the 83,444 assembled contigs, 15,314 were assigned to biological processes (27%), molecular functions (34%), and cellular components (39%). Within each of these three main categories, genes that annotated for translation, the nuclear-transcribed mRNA process, viral transcription, egg hatching, larval development, the response to drugs, protein biding, metal ion biding, and cytoplasm were the most abundant (Fig. 1). Important cell procedures related to early development were somewhat evidenced, such as with genes involved in cell motion, cell proliferation, cuticle formation, myogenesis, and locomotion.

Final functional classification and pathway assignment were performed using bi-directional BLAST with an E-value of 1e -3 against the KEGG database. Of these sequences, 16,213 had significant matches in the database. Among the matched sequences, metabolic pathways, such as carbon metabolism, the biosynthesis of amino acids, oxidative phosphorylation, glycolysis, the citrate cycle, and lipid metabolism, were well represented in *C. rogercresseyi* sequences. Given the important roles of lipids in the copepod lifecycle, especially during ecdysis, greater attention was placed on lipid metabolism. Genes were found in several pathways involved in fatty acid biosynthesis, such as fatty acid elongation, steroid biosynthesis, and ether lipid metabolism. Furthermore, genes related to nervous system development were highly annotated to signaling pathways such as the neuroactive ligand-receptor, the GABAergic and glutamatergic synapse, axon guidance, and the cholinergic synapse. Interestingly, immune response genes were found associated with the NF-kappa B signaling pathway, the TNF signaling pathway, and the Toll-like receptor, among others.

### Differentially expressed genes among developmental stages of *C. rogercresseyi*

In addition to obtaining gene annotations for the salmon louse, another major aim of the present transcriptomic study was to analyze the overall gene expression profile in order to identify genes participating in pivotal biological process and molecular functions related to the developmental stages, especially for larval stages and adult individuals. After *de novo* assembly, the contigs that showed matching reads for all samples were sorted to generate a gene reference dataset. Then, gene expression data was normalized for six RNA-seq experiments so as to separately compare the expression levels between larval stages, and female and male adult individuals. This approach was applied as the most critical physiological changes in the ontogeny of parasite copepods occur during the free-swimming (nauplius, copepodid), larval settlement (chalimus), and mature female and male adult phases [29]–[32].

**Figure 2:**
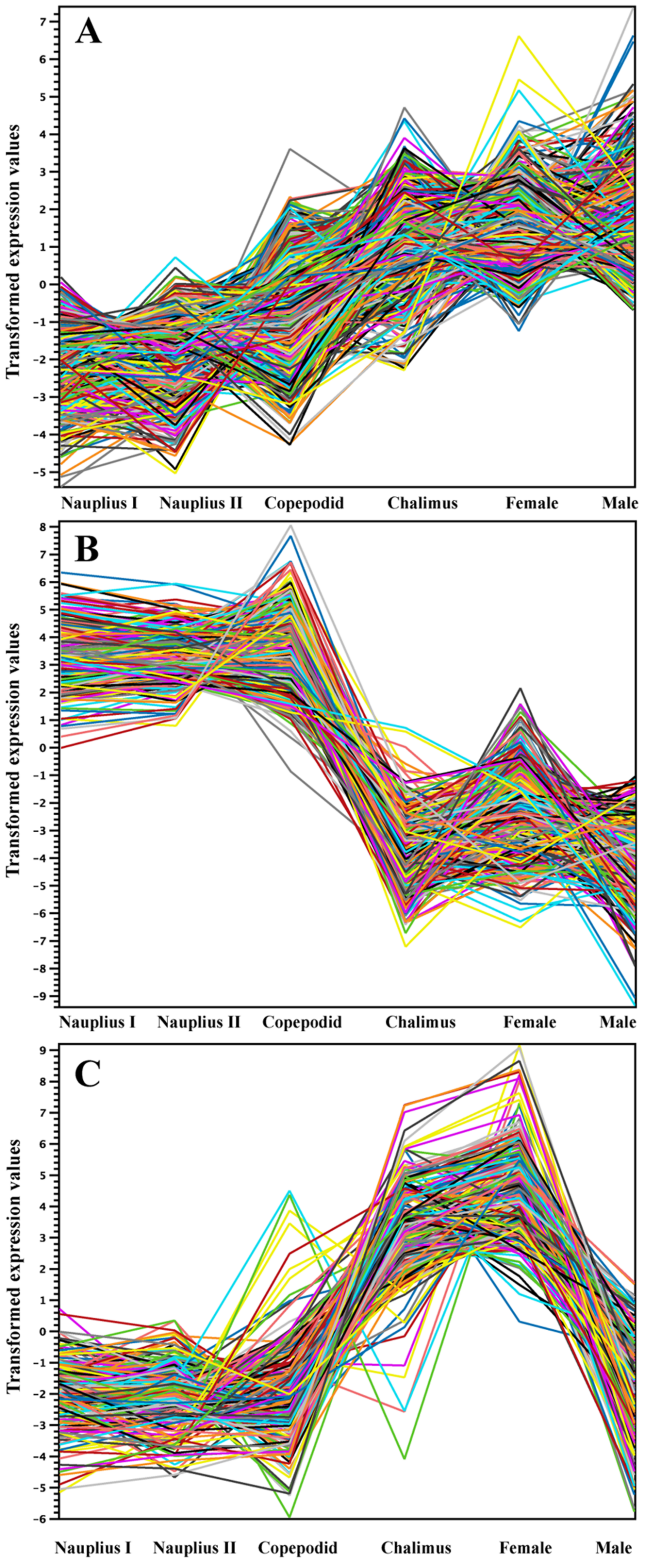
Gene expression profiles during larvae stages and adults of *C. rogercresseyi* generated by high-throughput transcriptome sequencing. (A) Overview of log_2_ expression ratios of all transcripts differentially expressed from nauplius I, nauplius II, copepodid, chalimus, adult females and adult males. (B and C) Two patterns of expression were detected by K-means algorithm using transformed expression values.

**Figure 3:**
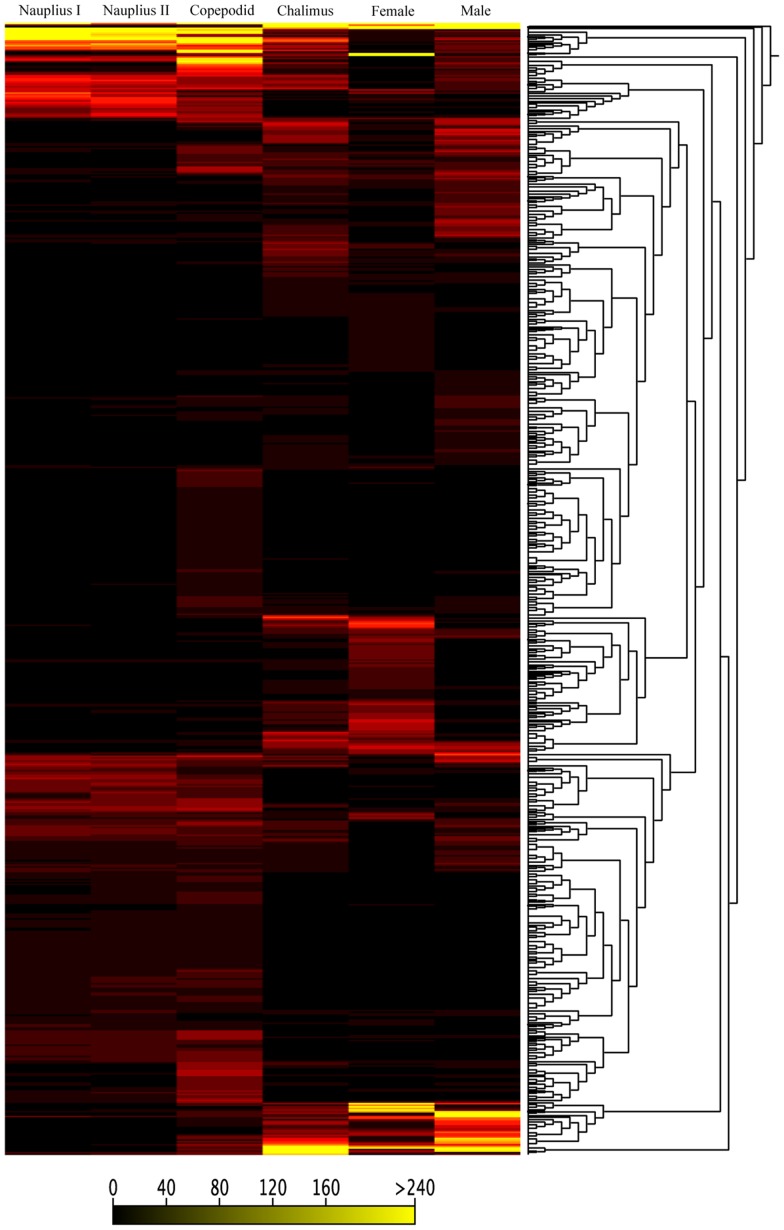
Heat map generated from *C. rogercresseyi* transcriptome data reflecting gene expression values in six developmental stages. Dendrograms of the transcription patterns were estimated for 83,444 contigs generated by de novo assembling. The bar color reflects the gene expression levels.

**Figure 4:**
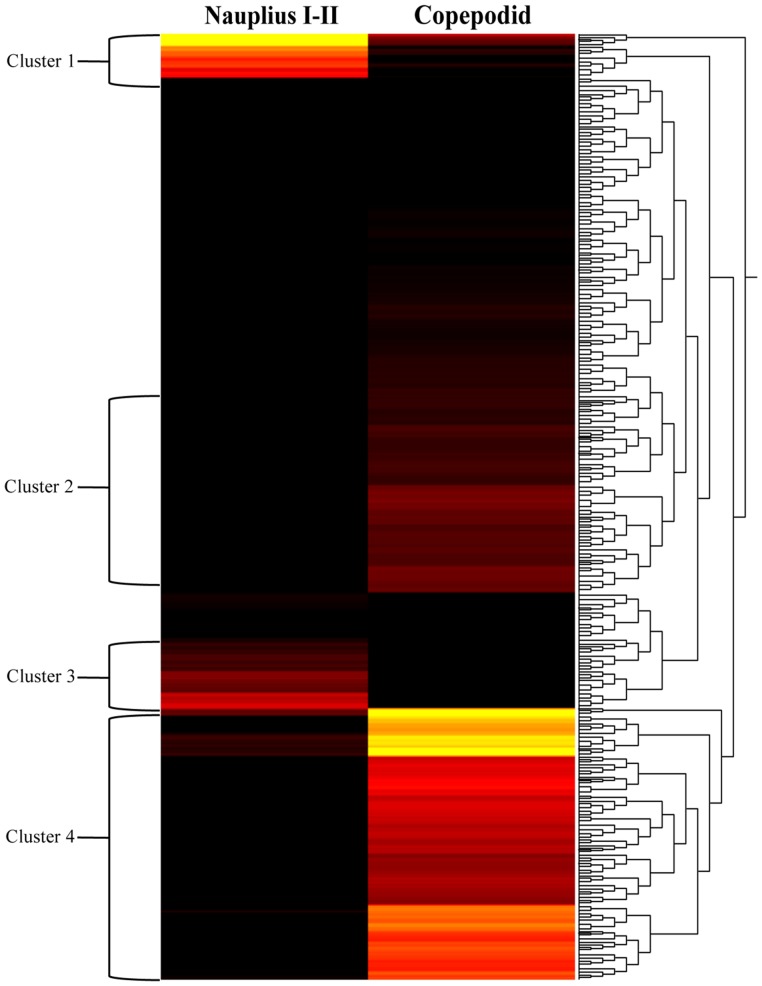
Clusters of gene expression levels between Nauplius I–II and copepodid stages of *C. rogercresseyi*. Dendrograms of the transcription patterns were estimated for 83,444 contigs generated by de novo assembling. The bar color reflects the gene expression level from black (low), red (medium) to yellow (high). Contig annotations of these 4 clusters are listed in Table S2.

**Figure 5:**
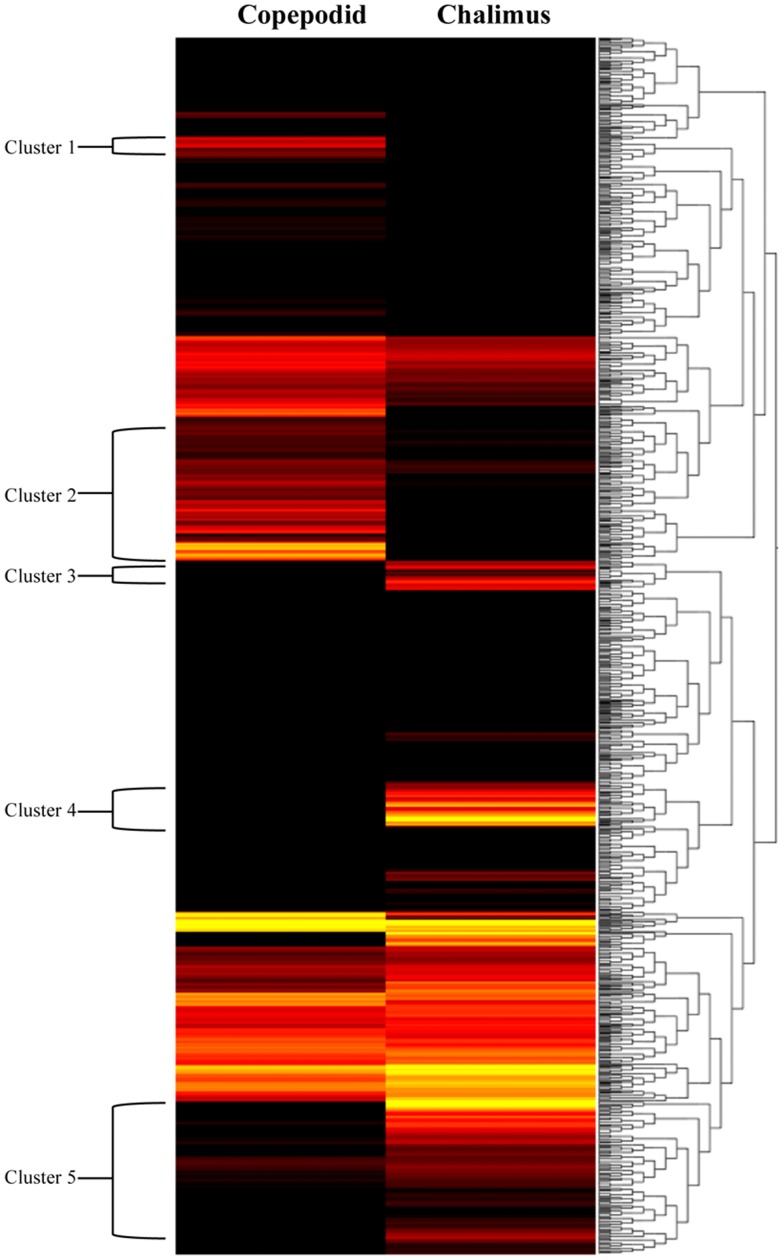
Clusters of gene expression levels between copepodid and chalimus stages of *C. rogercresseyi*. Dendrograms of the transcription patterns were estimated for 83,444 contigs generated by de novo assembling. The bar color reflects the gene expression level from black (low), red (medium) to yellow (high). Contig annotations of these 5 clusters are listed in Table S3.

**Figure 6:**
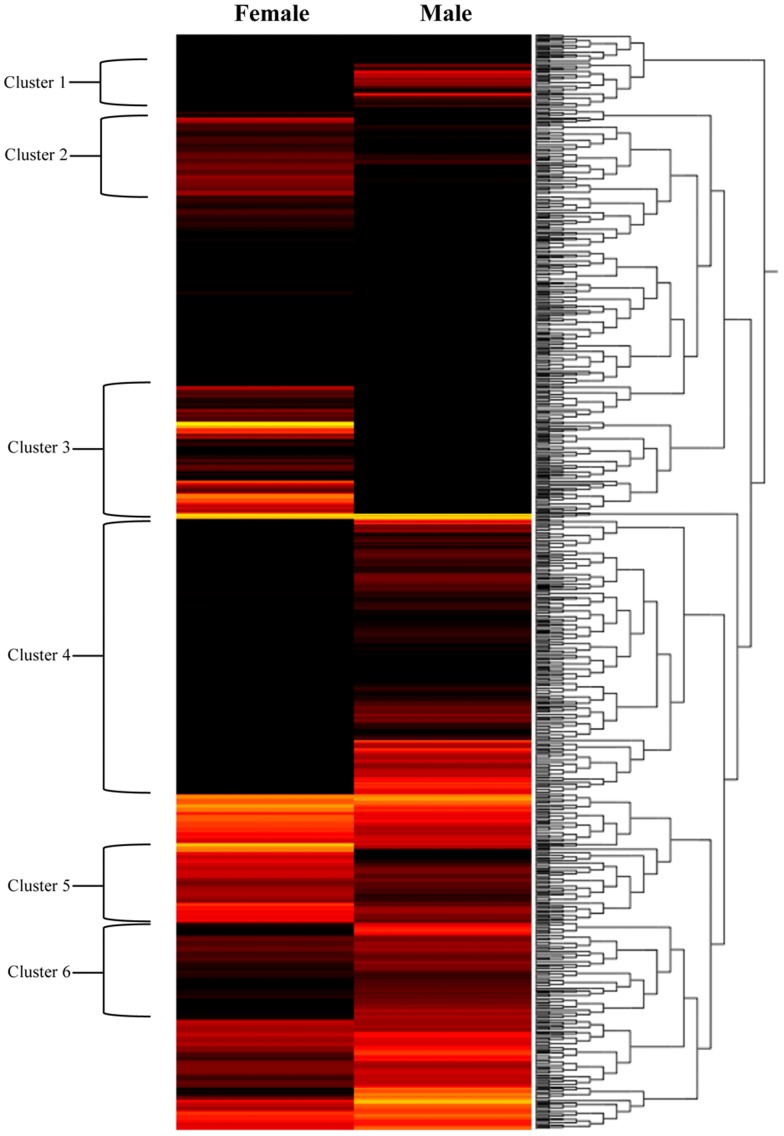
Clusters of gene expression levels between adult females and males of *C. rogercresseyi*. Dendrograms of the transcription patterns were estimated for 83,444 contigs generated by de novo assembling. The bar color reflects the gene expression level from black (low), red (medium) to yellow (high). Contig annotations of these 6 clusters are listed in Table S4.

Cluster analysis was conducted for 83,444 genes and showed differential transcription expression values among the analyzed developmental stages. The overall expression profiles are displayed in Figure 2. Clustering of the profiles from the six stages evidenced an increasing expression ratio (log_2_) from the nauplius I to adult stages at about a 5-fold change (Fig. 2A). However, high-resolution analysis of transcription patterns among the salmon lice stages revealed specific upregulated or downregulated gene clusters from the nauplius to adult stages. Herein, transcription activity was found associated with gene clusters showing up-regulation from the nauplius stage to the last developmental stages, as well as an down-regulation from early larval stages to male adults (Fig. 2B and C, respectively). Furthermore, the k-means and distance were estimated by the Manhattan method to identify clusters of candidate genes involved in specific gene expression patterns (Fig. 3). Through this, four clusters were observed differentially expressed between nauplius I–II and copepodid, where 604 transcripts (Fig. 4) were mainly overregulated in the copepodid stage (Clusters 4) and nauplius I–II stages (Clusters 1) (Table 2). It is important to note that no significant expression differences between nauplius I and nauplius II were observed. Then, for further analysis the two larval instars were considered as nauplius I–II stage. In addition, five clusters were evidenced differentially expressed between the copepodid and chalimus stages, where 2,426 transcripts (Fig. 5) were mainly associated to chalimus stage (Clusters 3, 4, and 5) as compared to copepodids (Clusters 1 and 2). The greatest differences in transcription expression were found in 271 putative genes that comprised Cluster 4 (Table 2). In addition, genes from female and male adults that showed differential transcription were highly identified into six clusters containing 2,478 transcripts (Fig. 6). Interestingly, half of the clusters that evidenced differential transcription activity were overregulated in females (Clusters 2, 3, and 5) as well as in the male transcriptome (Clusters 1, 4, and 6). Two clusters (3 and 5) linked to female gene expression displayed the highest RPKM values of the analyzed clusters (Table 2).

**Table 2:** Summary of clusters between Nauplius I–II/Copepodid, Copepodid/Chalimus, and Female/Male groups from *C. rogercresseyi* transcriptome.

	Number of sequences	% Annotated (<Evalue-5)	RPKM	
Clusters			Nauplius I–II	Copepodid
1	51	88,24	181.65	19.20
2	196	76,53	12.82	107.39
3	44	77,27	138.46	7.21
4	313	79,23	17.75	161.37
			Copepodid	Chalimus
1	215	60.93	96.33	6.44
2	880	73.98	163.17	28.06
3	182	61.54	3.43	154.18
4	271	62.73	2.85	422.72
5	878	78.82	38.25	106.35
			Female	Male
1	167	47.90	8.61	225.55
2	325	86.46	173.76	65.21
3	473	81.40	449.75	5.84
4	1022	68.59	33.46	196.03
5	273	65.20	431.26	152.29
6	218	81.19	88.07	190.24

In regards to gene annotation, relevant genes were identified through transcriptome cluster analysis between the nauplius I–II and copepodid. For instance, genes related to mitochondrial metabolism, and also to molting cycle were mainly associated to clusters 1, 2 and 3. In contrast, the cluster 4 evidenced a wide diversity of proteins, including an important number of hypothetical proteins annotated for *Lepeophtheirus salmonis* and *Daphnia pulex*. Moreover, for copepodid and chalimus stages of *C. rogercresseyi*, clusters 1, 3, and 6 were comprised of genes related to nervous system development, such as the neuronal acetylcholine receptor subunit alpha-3, Cerebellin-3, High-affinity choline transporter, and GABA-alpha subunit. Clusters 2 and 4 were mainly annotated to genes associated with cuticle and contractile elements, such as the cuticle protein, ferritin, myosin and actin, and with some genes related to the immune response, such as akirin, agglutinin isolectin, E-selectin, peroxinectin, and cathepsin. In addition, clustering analysis between female and male adults revealed a major diversity of identified genes. Some genes were linked to the morphogenesis process and cellular proliferation, such as the cuticle protein 6, gamma-crystallin A, hemicentin protein, calreticulin, and vasa gene (Clusters 1–3). Genes involved in the processes of gametogenesis and reproduction, such as the proliferation-associated protein, insulin-like growth factor-binding protein, nuclear sperm protein, vitellogenin, and estradiol 17-beta-dehydrogenase, were also annotated (Clusters 4–6). A detailed list of relevant, identified genes is shown in the supplementary material (Table S3–S5 in File S2)

In order to identify the genes highly expressed between copepodid and chalimus stages, and between female and male adults, statistical analyses visualized on a Volcano plot were performed to evaluate fold change values (Fig. S1 in File S1). From this, genes associated with Gene Ontology terms such as amino acid transfer, repair and breakdown, metabolism, and nervous system development were identified for the nauplius I–II, copepodid and chalimus stages. An upregulation specific to the copepodid stage was observed for the genes metalloproteinase, arginine kinase, E-selectin, L-selectin, tropomyosin, cuticle proteins, flotillin, allotostatin, and opsin, among others. For the chalimus stage, the genes trypsin, alpha amylase, carboxipeptidase, bleomycin, gamma-crystalin A, nanos homolog, and vitellogenin were overregulated (Table 3 and 4). With regards to female and male adults, most candidate genes were related to cytoskeletal and contractile elements, reproduction, cell development, morphogenesis, and the transcription and translation process. For female adults, hemicentin, TSP1-containing protein, vitellogenin, homeobox, vasa, argonaute, and several transcription factors were upregulated. For salmon louse males, actin, troponin, myosin, cuticle protein, brain-specific angiogenesis inhibitor and sperm proteins such as nuclear autoantigenic sperm protein, motile sperm domain-containing protein 1 and peroxidosomal N1-acetyl-spermine/spermidine oxidase were mainly overregulated (Table 5). Finally, statistical analysis showed 63, 166 and 114 hypothetical proteins and unannotated contigs up/down-regulated from nauplius I–II/copepodid, copepodid/chalimus and female/male adults, respectively (Fig. S2 in File S1). A detailed list of hypothetical proteins and unannotated contigs is shown in the supplementary material (Table S6–S8 in File S3).

**Table 3:** Genes showing differential transcription expression between Copepodid/Nauplius I–II groups.

Feature ID	Lowest E-value	LOG2 (fold change)	LOG10 (p-value)	Annotation
				***Aminoacid transfer, repair and breakdown***
contig67130	8,57139E-35	7,1	11,5	Carbonic anhydrase 1 [Caligus rogercresseyi].
contig67131	1,13531E-53	8,7	9,2	Carbonic anhydrase 1 [Caligus rogercresseyi].
contig67168	6,5465E-39	7,3	7,0	Carbonic anhydrase 1 [Caligus rogercresseyi].
contig8626	2,8566E-103	6,9	7,4	Ascorbate peroxidase precursor [Caligus rogercresseyi].
contig83793	1,91917E-24	3,9	7,7	Arginine kinase 1 [Neocaridina denticulata].
contig19878	2,62427E-07	8,5	8,3	Metalloproteinase [Lepeophtheirus salmonis].
contig21633	5,11652E-11	8,6	10,3	Metalloproteinase [Lepeophtheirus salmonis].
contig37955	4,23278E-17	9,5	15,1	Metalloproteinase [Lepeophtheirus salmonis].
contig38246	2,13864E-48	5,4	9,7	Metalloproteinase [Lepeophtheirus salmonis].
contig4456	1,37039E-48	5,4	13,3	Metalloproteinase [Lepeophtheirus salmonis].
contig4457	1,30919E-23	8,3	6,9	Metalloproteinase [Lepeophtheirus salmonis].
contig49660	5,09513E-39	4,2	13,1	Metalloproteinase, partial [Lepeophtheirus salmonis].
				***Cytoskeletal and contractile elements***
contig74472	2,98803E-15	6,8	7,2	Myosin light chain, partial [Artemia franciscana].
contig49321	1,53252E-27	6,0	11,2	Troponin C, isoform 1 [Caligus rogercresseyi].
contig9694	1,05829E-75	4,4	15,4	Troponin C, isoform 1 [Caligus rogercresseyi].
				***Molting cycle***
contig32752	2,32173E-30	8,1	7,6	Cuticle protein 18.6, isoform B [Caligus clemensi].
contig46463	4,72236E-24	6,6	7,1	Cuticle protein 18.6, isoform B [Caligus clemensi].
contig16836	1,21928E-34	6,5	12,7	Cuticle protein 6 [Lepeophtheirus salmonis].
contig26826	3,10846E-35	4,8	10,3	Cuticle protein 6 [Lepeophtheirus salmonis].
contig48959	1,53073E-24	4,1	15,7	Cuticle protein 6 [Lepeophtheirus salmonis].
contig53248	9,42643E-44	5,2	13,5	Cuticle protein 7 [Lepeophtheirus salmonis].
contig63924	2,35417E-28	4,6	9,5	Pupal cuticle protein 20 precursor [Lepeophtheirus salmonis].
contig18404	1,53294E-12	5,2	10,6	Putative cuticle protein [Lepeophtheirus salmonis].
contig32517	2,27674E-52	5,6	10,0	Putative cuticle protein [Lepeophtheirus salmonis].
contig36786	0,00254215	5,6	15,2	Putative cuticle protein [Lepeophtheirus salmonis].
contig37317	0,00820674	6,9	9,0	Putative cuticle protein [Lepeophtheirus salmonis].
contig45391	7,48002E-52	4,4	9,2	Putative cuticle protein [Lepeophtheirus salmonis].
contig4994	2,2983E-55	6,3	14,9	Putative cuticle protein [Lepeophtheirus salmonis].
contig56065	1,10838E-52	4,0	9,7	Putative cuticle protein [Lepeophtheirus salmonis].
contig47416	2,66152E-23	5,6	15,7	Putative cuticle protein, partial [Lepeophtheirus salmonis].
contig4995	8,52173E-35	7,4	11,8	Putative cuticle protein, partial [Lepeophtheirus salmonis].
				***Metabolism, homeostasis, mitochondrial genes***
contig47377	7,86399E-11	5,9	7,3	ATP synthase subunit d, mitochondrial [Caligus rogercresseyi].
contig3896	1,25062E-42	6,3	7,0	NADH dehydrogenase subunit 3 (mitochondrion) [Caligus rogercresseyi].
contig1025	7,35293E-48	−6,7	10,3	NADH dehydrogenase subunit 5 (mitochondrion) [Caligus rogercresseyi].
contig34749	2,7007E-123	4,0	15,4	NADH dehydrogenase subunit 5 (mitochondrion) [Caligus rogercresseyi].
contig22665	1,16544E-23	8,4	8,8	BCS-1-like protein [Lepeophtheirus salmonis].
contig5905	3,8909E-102	6,3	11,9	Cytochrome b (mitochondrion) [Lepeophtheirus salmonis].
contig729	2,0278E-180	6,2	10,0	Cytochrome b (mitochondrion) [Lepeophtheirus salmonis].
contig22034	8,75655E-37	8,2	7,4	Cytochrome c oxidase subunit I (mitochondrion) [Caligus clemensi].
contig44642	7,09936E-14	5,6	10,9	Cytochrome c oxidase subunit I (mitochondrion) [Caligus clemensi].
contig2046	4,15251E-94	8,1	7,0	Cytochrome c oxidase subunit I (mitochondrion) [Caligus clemensi].
contig2066	2,10069E-62	6,5	9,0	Cytochrome c oxidase subunit I (mitochondrion) [Caligus clemensi].
contig19297	1,27201E-98	6,8	9,9	Cytochrome c oxidase subunit I (mitochondrion) [Caligus clemensi].
				***Reproduction***
contig41083	3,04598E-05	4,3	10,9	vitellogenin-like protein [Lepeophtheirus salmonis].
				***Transcription and translation***
contig40719	8,30837E-16	4,8	7,9	40S ribosomal protein S26 [Lepeophtheirus salmonis].
				***Other***
contig3253	4,09671E-38	5,2	8,1	Cathepsin L1 precursor [Caligus clemensi].

**Table 4:** Genes showing differential transcription expression between Chalimus/Copepodid groups.

Feature ID	Lowest E-value	LOG2 (fold change)	LOG10 (p-value)	Annotation
				***Aminoacid transfer, repair and breakdown***
contig55728	5,63172E-48	−7,4	8,3	Carbonic anhydrase 1 [Caligus rogercresseyi].
contig13342	1,20771E-09	−6,0	8,8	Histone-lysine N-methyltransferase [Lepeophtheirus salmonis].
contig16041	7,2939E-153	−6,6	11,3	Arginine kinase [Lepeophtheirus salmonis].
contig548	9,1455E-147	−6,1	10,6	Adenosylhomocysteinase [Lepeophtheirus salmonis].
contig14750	2,50162E-87	−4,8	10,3	Prohormone-4-like protein [Acartia pacifica].
contig41086	5,03513E-48	5,3	8,0	Trypsin-1 [Caligus rogercresseyi].
contig42305	7,32018E-71	6,1	8,2	Trypsin-1 [Caligus rogercresseyi].
contig67172	1,34774E-44	−8,7	10,2	Metalloproteinase [Lepeophtheirus salmonis].
contig27850	1,11517E-14	5,8	12,3	Metalloproteinase [Lepeophtheirus salmonis].
contig8942	0	6,5	8,5	Alpha-amylase A precursor [Caligus rogercresseyi].
contig33626	2,32259E-32	7,0	8,8	Trypsin-1 [Caligus rogercresseyi].
contig11498	2,24443E-80	6,9	8,6	Carboxypeptidase B [Caligus rogercresseyi].
contig46635	8,8318E-121	9,5	15,0	Bleomycin hydrolase [Caligus rogercresseyi].
				***Cellular process***
contig6529	5,7491E-135	−7,6	13,1	L-selectin [Lepeophtheirus salmonis].
contig16212	4,21268E-80	−5,6	8,8	E-selectin precursor [Lepeophtheirus salmonis].
				***Cytoeskeletal and contractile elements***
contig19095	5,31585E-23	−9,3	14,2	Tropomyosin-2 [Caligus rogercresseyi].
contig6216	0	−9,1	12,5	Myosin heavy chain isoform 3 [Daphnia pulex].
contig46434	1,25135E-07	−8,5	8,6	Kelch-like protein 14 [Gallus gallus]
contig10586	4,7262E-121	−8,2	13,6	PDZ and LIM domain protein 3 [Lepeophtheirus salmonis].
				***Molting cycle***
contig50594	1,50909E-10	−9,1	12,7	Putative cuticle protein, partial [Lepeophtheirus salmonis].
contig63924	2,35417E-28	−9,0	12,1	Pupal cuticle protein 20 precursor [Lepeophtheirus salmonis].
				***Metabolism, homeostasis, mitochondrial genes***
contig33120	4,25794E-09	−9,2	13,5	Beta-carotene 15,15′-monooxygenase [Gallus gallus]
contig2959	0	−8,1	6,9	Sodium-potassium ATPase alpha subunit [Eriocheir sinensis].
contig15241	1,02987E-57	−6,0	9,1	Calmodulin [Caligus rogercresseyi].
contig32254	1,40283E-49	−5,4	11,1	Calmodulin [Electrophorus electricus]
contig19809	3,90075E-69	−5,6	7,7	Zinc finger protein rotund [Drosophila melanogaster]
contig28691	1,18912E-15	−8,2	7,4	BCS-1 protein, partial [Lepeophtheirus salmonis].
contig28707	1,93422E-15	−5,7	11,3	BCS-1 [Amphibalanus amphitrite].
contig11399	1,35737E-41	−6,2	6,8	Diuretic hormone class 2 [Lepeophtheirus salmonis].
contig23270	7,05676E-58	8,2	14,0	Elongation of very long chain fatty acids protein [Caligus clemensi].
				***Neuronal involvement and nervous system***
contig14064	0	−4,3	9,5	Nicotinic acetylcholine receptor subunit alpha 3 [Pandalopsis japonica].
contig30958	0	−7,7	9,7	Flotillin-1 [Drosophila melanogaster]
contig26943	8,3871E-107	−6,2	10,4	Synaptotagmin 1 protein, variant 1 [Daphnia pulex].
contig16211	4,4869E-118	−5,1	11,9	Synaptotagmin 1[Drosophila melanogaster].
contig21056	6,4435E-160	−5,4	9,6	Flotillin-1 [Drosophila melanogaster].
contig21663	3,93537E-11	−5,7	11,8	Allatostatin precursor protein [Panulirus interruptus].
contig40313	1,24728E-47	−7,5	8,9	Protein TKR [Drosophila melanogaster]
contig36359	5,9044E-112	−5,6	7,7	Frequenin-1 [Drosophila melanogaster]
contig14962	4,2039E-167	−4,5	11,6	Opsin, partial [Tigriopus californicus].
contig51504	3,98118E-28	5,2	8,4	TSP1-containing protein [Lepeophtheirus salmonis].
contig28899	2,6935E-16	5,7	11,6	Gamma-crystallin A [Lepeophtheirus salmonis].
contig37761	1,04894E-13	6,4	7,8	Gamma-crystallin A [Lepeophtheirus salmonis].
contig27330	6,62807E-29	6,9	8,7	Gamma-crystallin A [Lepeophtheirus salmonis].
				***Reproduction***
contig27769	4,06923E-79	5,7	14,8	Vitellogenin-like protein [Lepeophtheirus salmonis].
contig4055	6,6744E-134	5,7	8,1	Nanos homolog 2 [Caligus rogercresseyi].
contig40296	1,61825E-76	6,9	13,8	Vitellogenin-like protein [Lepeophtheirus salmonis].
contig83298	4,20765E-45	8,1	13,1	Vitellogenin 2 [Lepeophtheirus salmonis].
				***Transcription and translation***
contig4248	1,25028E-46	−4,4	9,1	Sox14 protein [Scylla paramamosain].
contig16105	5,65021E-54	−7,0	9,1	Dachshund-like protein [Daphnia pulex].
contig1801	7,76295E-86	5,9	8,2	RNA-binding protein Musashi homolog 2 [Caligus clemensi].
				***Other***
contig46734	2,21072E-42	−8,7	9,8	Hypothetical protein [Lepeophtheirus salmonis].
contig22388	5,65549E-69	−5,5	8,1	Non-symbiotic hemoglobin 1 [Caligus clemensi].
contig5412	2,3542E-144	−5,0	8,4	Cathepsin L precursor [Caligus rogercresseyi].
contig510	2,38835E-47	8,4	8,2	Mitotic apparatus protein p62 [Caligus rogercresseyi].
contig31747	0	8,6	9,5	Cytosolic non-specific dipeptidase [Caligus rogercresseyi].

**Table 5:** Genes showing differential transcription expression between Male/Female groups.

Feature ID	Lowest E-value	LOG2 (fold change)	LOG10 (p-value)	Annotation
				***Aminoacid transfer, repair and breakdown***
contig3464	2,36753E-14	−6,0	10,2	Acetoacetyl-CoA synthetase [Caligus rogercresseyi].
contig11157	4,5629E-106	−5,7	7,4	Quinone oxidoreductase [Lepeophtheirus salmonis].
contig10732	1,7976E-130	−5,2	10,3	Carboxypeptidase B [Lepeophtheirus salmonis].
contig2325	1,25183E-80	−4,5	11,1	Protein disulfide-isomerase 2 precursor, partial [Lepeophtheirus salmonis].
contig44329	1,32425E-37	6,6	13,1	Arginine kinase [Lepeophtheirus salmonis].
contig21833	5,83789E-15	5,7	9,7	Serine protease 33 precursor [Lepeophtheirus salmonis].
				***Cellular process***
contig915	1,46311E-43	−7,9	11,0	Transmembrane protein nessy [Caligus clemensi].
contig9286	0	−6,2	7,2	Transmembrane protein 20 [Caligus rogercresseyi].
				***Cytoskeletal and contractile elements***
contig24790	5,4357E-108	4,7	8,7	Actin [Lepeophtheirus salmonis].
contig83837	7,06506E-17	4,9	12,5	Troponin T [Lepeophtheirus salmonis].
contig61222	3,9463E-132	5,7	12,7	Tropomyosin [Caligus clemensi].
contig47063	2,07041E-18	5,0	10,1	Myosin heavy chain type 3, partial [Penaeus monodon].
contig43406	2,82083E-46	8,2	7,4	Beta-actin, partial [Palaemonetes pugio].
contig7293	7,34563E-51	7,7	9,7	Four and a half LIM domains protein 2 [Lepeophtheirus salmonis].
contig54932	2,43576E-40	7,8	10,8	Chymotrypsin-2 [Lepeophtheirus salmonis].
				***Molting cycle***
contig44524	3,19226E-32	8,4	8,1	Pupal cuticle protein 20 [Lepeophtheirus salmonis].
contig56379	1,21557E-64	9,4	15,4	Putative cuticle protein [Lepeophtheirus salmonis].
contig19428	1,92267E-26	6,4	15,7	Putative cuticle protein [Lepeophtheirus salmonis].
				***Metabolism, homeostasis, mitochondrial genes***
contig26741	8,2065E-114	5,0	8,3	Aquaporin-3 [Lepeophtheirus salmonis].
contig34541	2,11208E-23	5,2	13,1	BCS-1-like protein [Lepeophtheirus salmonis].
				***Neuronal involvement and nervous system***
contig6471	3,09208E-35	−8,8	10,7	Hemicentin-1 [Homo sapiens]
contig13628	8,44815E-20	−8,2	7,3	Hemicentin protein, partial [Lepeophtheirus salmonis].
contig36528	2,26484E-23	−6,1	8,1	TSP1-containing protein, partial [Lepeophtheirus salmonis].
contig52422	4,45343E-14	8,3	14,8	Brain-specific angiogenesis inhibitor 1 [Homo sapiens]
				***Reproduction***
contig21011	6,56675E-12	−9,4	16,0	vitellogenin 2 [Lepeophtheirus salmonis].
contig23903	1,50955E-71	−9,4	15,7	Vitellogenin 2 [Lepeophtheirus salmonis].
contig34688	5,29891E-29	−9,3	14,4	vitellogenin 2 [Lepeophtheirus salmonis].
contig25754	1,51763E-42	−9,2	13,7	Vitellogenin 1 [Lepeophtheirus salmonis].
contig21448	5,50959E-40	−9,2	13,4	Vitellogenin 2 [Lepeophtheirus salmonis].
contig777	1,08639E-40	−8,9	11,4	Estradiol 17-beta-dehydrogenase [Xenopus tropicalis]
contig256	1,09674E-19	5,6	9,3	Nuclear autoantigenic sperm protein [Penaeus monodon].
contig8354	1,6416E-100	8,6	9,1	Motile sperm domain-containing protein 1 [Caligus rogercresseyi].
contig14073	7,8447E-148	6,3	14,0	Peroxisomal N1-acetyl-spermine/spermidine oxidase [Caligus rogercresseyi].
				***Cell develpment and morphogenesis***
contig2604	3,31853E-48	−6,8	8,1	Homeobox protein Ht-En [Caligus clemensi].
contig1263	2,23329E-71	−4,5	9,4	Vasa [Lepeophtheirus salmonis].
contig5709	1,81336E-98	−8,6	9,2	Argonaute 1 isoform C [Marsupenaeus japonicus].
contig13230	7,3186E-152	−6,2	8,3	Cell division protein kinase 4 [Caligus clemensi].
contig4147	1,25397E-15	−6,1	11,0	Centromere-associated protein [Mus musculus]
contig17540	4,33246E-31	−6,2	13,0	Histone H4 [Caligus rogercresseyi].
contig56079	1,37621E-20	5,2	15,7	Sarcoplasmic calcium-binding protein, beta chain [Caligus rogercresseyi].
contig8725	7,7017E-102	5,7	13,9	Glycoprotein G precursor [Caligus rogercresseyi].
				***Transcription and translation***
contig3246	6,30674E-40	−9,0	11,7	Putative exonuclease I [Lepeophtheirus salmonis].
contig6559	4,30672E-71	−8,2	13,2	60S ribosomal protein L35 [Caligus rogercresseyi].
contig2546	2,26515E-46	−8,1	13,0	Putative SPT transcription factor family member [Lepeophtheirus salmonis].
contig12157	2,49441E-64	−7,9	11,3	Spz3 [Litopenaeus vannamei].
contig4045	8,93417E-68	−5,0	11,6	Proliferation-associated protein 2G4 [Lepeophtheirus salmonis].
contig9121	1,17798E-82	−7,4	7,9	Argonaute 1 isoform C [Marsupenaeus japonicus].
contig45438	9,76658E-17	−7,1	12,4	Ubiquitin [Caligus rogercresseyi].
contig7577	5,51092E-50	−6,4	9,5	40S ribosomal protein S4 [Lepeophtheirus salmonis].
contig55998	4,27953E-28	7,3	7,8	Elongation factor 1-alpha [Lepeophtheirus salmonis].
				***Other***
contig3827	3,40176E-26	−9,4	16,0	Hypothetical protein DAPPUDRAFT_323318 [Daphnia pulex].
contig42870	4,3447E-14	9,1	12,9	Venom allergen [Vespa crabro]

In overall, the gene expression patterns revealed through the developmental stages of *C. rogercresseyi*, suggest lower changes of transcription activity between nauplius I, II and copepodid stages. In contrast, higher gene expression differences were found during the infective stage of chalimus and adults of the salmon louse. The principal component analysis showed correlation values that grouped nauplius I, nauplius II and copepodid stages by separate from chalimus and adults instars (Fig. S3 in File S1). Moreover, to confirm the usefulness of the *C. rogercresseyi* cDNA database established by the Illumina paired-end sequencing method, we investigated by qRT-PCR the expression of 9 genes selected from catalytic activity, nervous system development, molting, contractile elements, reproduction and cellular process (Fig. S4–S8 in File S1). The correlation between expression levels quantified by qPCR and the *in silico* analysis confirmed the robustness of the illumina sequencing results (Fig. S9 in File S1)

**Figure 7:**
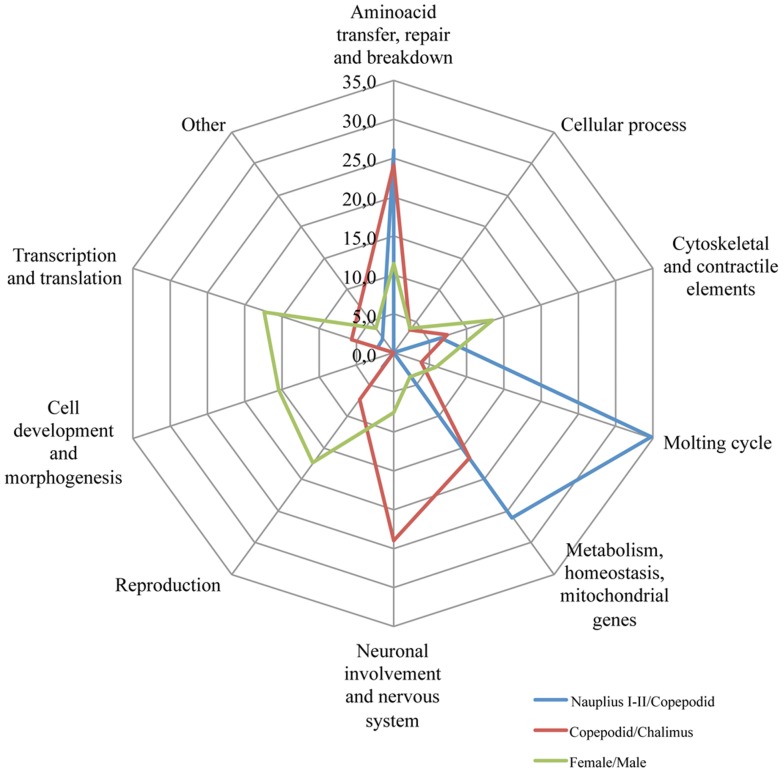
Radar plot of contigs with significant expression values (P≤10–16; |fold-change|>5) in terms of percentages for nauplius I–II/copepodid, copepodid/chalimus and female/male from *C. rogercresseyi*. Annotated contigs were associated to (i) aminoacid transfer, repair and breakdown, (ii) cellular process, (iii) cytoskeletal and contractile elements, (iv) molting cycle, (v) metabolism, homeostasis, mitochondrial genes, (vi) neuronal involvement and nervous system, (vii) reproduction, (viii) cell development and morphogenesis, (ix) Transcription and translation and (x) others.

Radar plot of contigs with significant expression values (P≤10–16; |fold-change|>5) in terms of percentages for nauplius I–II/copepodid, copepodid/chalimus and female/male from *C. rogercresseyi* were analyzed in order to evidenced the proportions of genes up/down-regulated that are associated to key biological process and molecular functions (Fig. 7). Interestingly, the analysis revealed that the nauplius I–II, copepodid and chalimus stages are mainly annotated to aminoacid transfer/repair/breakdown, metabolism, molting cycle, and nervous system development. Additionally, genes showing differential transcription in female and male adults were highly related to cytoskeletal and contractile elements, reproduction, cell development, morphogenesis, and transcription-translation processes.

## Discussion

Sea lice are the most prevalent ectoparasites found in the farmed salmon industry worldwide, and two species, *Lepeophtheirus salmonis* (Krøyer, 1838) and *Caligus rogercresseyi* (Boxshall and Bravo, 2000), are responsible for major economic losses in countries such as Norway, Scotland, Canada, and Chile [33]. According to Hamre et al. [34], the complete life cycle is now known for 17 species of Caligidae, as represented by just three genera, *Caligus* (12 species), *Lepeophtheirus* (four species), and *Pseudocaligus* (one species). However, the number of developmental stages appears to vary among species, with the free-living phase being comprised of two nauplii stages and the infective copepodid stage, while there are several chalimus and adult stages [35]. In *Caligus* species, four chalimus instars have been found, the last of which molts into the definitive adult [24], [25], [36], [37]. In contrast, the life cycle of *Lepeophtheirus* species have been reported to have four chalimus and two pre-adult stages that allow the louse the ability to detach from a temporary frontal filament shortly after molting and move over the surface of the skin [38]. However, recent findings from observing chalimus larvae molting and through morphometric cluster analysis from *L. salmonis* reported only two chalimus stages, and, consequently, a life cycle comprised of six post-nauplius instars [34].

Understanding salmon louse biology is critical for establishing strategies that allow for the control and management of this ectoparasite. However, evidence supporting morphological and physiological changes in correlation with transcriptome profiles during the life cycle of salmon lice is still limited. For instance, EST collections for the developmental stages of *L. salmonis* have only been reported in female and male adults [31], [32], [39], which so far represents the most comprehensive and publicly available transcriptome for *L. salmonis* at 129,250 transcripts [23]. In this context, the present study provides 84,023 high quality contigs and, subsequently, 29,000 significant annotated proteins from different developmental stages of the salmon louse *C. rogercresseyi*. This sequencing effort represents the most comprehensive transcriptome resource available for this caligid species.

Salmon lice included in the present RNA-seq study were evaluated between the nauplius I–II and copepodid, copepodid and chalimus stages, and also between female and male adults. This approach was applied in order to identify relevant transcriptome profiles across larvae instars and with sexual differentiation. In fact, it could be hypothesized that these developmental phases are representative of the major physiological changes during the life cycle of copepods. For instance, a study related to peptidergic signaling in *C. finmarchicus* reported that the highest expression levels from six stages (embryo, early nauplius, late nauplius, early copepodid, late copepodid, and adult) are seen in the naupliar and copepodid stages, while the lowest levels are present in embryos and adult females [29]. Specifically in the copepodid stage, host-seeking behavior has been displayed by *L. salmonis* during its infectious stage, including moving towards river mouths and maintaining location in haloclines during salmon migrations [40]. Consequently, the copepod must be able to cope with abiotic stress conditions [32] and respond to the inflammatory defense mechanisms at the site of salmon parasite attachment [41]. Furthermore, relatively little is known concerning sex differentiation and its endocrine control in crustaceans, and most available data have been obtained in decapods [42]. However, transcriptome sequencing studies have facilitated the discovery of novel sex-related genes, which thus far have suggested pivotal transcriptional differences between female and male adults [31], [43].

The present transcriptome analysis of *C. rogercresseyi* revealed 3,030 transcripts that comprised nine clusters, which were differentially expressed between the nauplius I–II, copepodid and chalimus stages. Interestingly, some upregulated genes were mainly associated with metalloproteinase, arginine kinase, and cuticle protein, which evidence participation in the digestion of intake proteins, tissue development, cuticle remodeling, and in specific cleavage events to activate or inactivate proenzymes and bioactive peptides [44]. With respects to nervous system development in copepodids, some relevant genes such as nicotinic acetylcholine receptor, flotillin, synaptotagmin, allotostatin, frequenin, and opsin were highly overexpressed. These results are congruent with previous studies of transcriptome profiles reported for copepodid stages [29], [45], [46]. Furthermore, a study by Wilson and Hartline [47] demonstrated high peripheral and central nervous system development in individuals transitioning from the nauplius to copepodid stage. The upregulation of allotostatin could be associated with the regulation of juvenile hormone production, or, more interestingly, with recent findings where the activation of neurons, or neuroendocrine cells, that expressed the neuropeptide allotostatin modulated feeding behavior in *Drosophila*, including increased food intake and enhanced behavioral responsiveness to nutrients or molecular clues [48]. It is important to note that these effects on feeding behavior could be related to changes induced by the start of the parasitic phase in the salmon louse. Furthermore, investigations of opsin function outside of vertebrate systems have long been focused on arthropod visual pigments [49], indicating that copepods possess a sensory apparatus sensitive to different wavelengths that could have implications during the host-finding process, especially in the copepodid stage [50].

For the chalimus stage, trypsin, alpha amylase, and carboxipeptidase genes were overregulated. Peptidases from the different families may be involved in a wide range of cellular and biological processes, thus making it difficult to infer specific functions across salmon louse development. Host blood has been reported as a major food component for the salmon louse *L. salmonis* [51]. Blood degradation in several hematophagus organisms has been shown to require the catalysis of several peptidases [52], [53]. Of the regulated peptidases in the present study, the most overregulated was trypsin, a secretory endopeptidase within the serine protease superfamily. This superfamily includes important digestive enzymes that constitute a major part of digestive fluids and act as activators of other digestive enzymes. These results are congruent with previous reports on the interaction between parasitic copepods and salmon hosts [54], [55].

In addition, genes showing differential transcription from female and male adults were highly annotated into six clusters comprised of 2,478 transcripts. For female adults, sex-related genes such as vitellogenins and estradiol 17-beta-dehydrogenase were identified. However, effects of the vertebrate-like steroid hormones on reproductive processes, such as oocyte maturation in crustaceans, still remain unresolved. Vitellogenins are the major yolk proteins in most invertebrates, and several different vitellogenins typically give rise to vitelline granules in mature eggs [56]. The role of multiple vitellogenin genes in some organisms, such as insects, is unknown [57] despite that proteins with domain structures similar to vitellogenins are also involved in other developmental processes, such as in the regulation of osmolarity, immunity, and clotting [58]. The present data showed a wide diversity of vitellogenins, including LsVit1 and LsVit2 as reported in *L. salmonis* [59], and several vitellogenin-likes proteins. Moreover, genes associated with cell development, including homologues of vasa, homeobox, argonaute, cell division protein kinase, and centromere-associated protein, were also specifically expressed in female adults. For salmon louse males, transcription activity related with cytoskeletal and molting cycle, as well as with sperm proteins were mainly overregulated. Based on the data of the present study it is therefore likely that the actin, troponin, myosin, and cuticle proteins are an important part of cuticle formation during the final molt for *C. rogercresseyi*. The sex-related genes reported in the present study represent novel molecular information regarding salmon louse reproduction.

The initial analysis of *C. rogercresseyi* transcriptome revealed that approximately 71.4% had no significant hits in GenBank using the nr-database. Even the re-annotation of the contigs revealed a total of 13% novel homologous proteins to *L. salmonis*. Similar high proportions of novel genes have been reported in non-model crustacean species [20], [43]. Furthermore, a total of 230 hypothetical proteins evidenced significant gene expression differences among the developmental stages, demonstrating the potential for discovery of unknown genes and novel biological processes involved in the life cycle of salmon lice.

## Conclusions

The present study represents a step forward in identifying a number of possible conserved genes that are likely to be involved in various important biological activities. Using *de novo* assembly, 83,444 high quality contigs and 24,000 genes, as based on known proteins, were identified from the *C. rogercresseyi* transcriptome. Future studies will address validating the discovered gene profiles, thus avoiding misinterpretations of the functional genomics information. The present data provide the most comprehensive transcriptome resource available for *C. rogercresseyi*, which should be used for future genomic studies linked to host-parasite interactions.

## Supporting Information

Table S1 **Primer list for qPCR validated in** ***C. rogercresseyi*** **genes.**
(DOCX)

Table S2 **BLASTx results from** ***C. rogercresseyi*** **transcriptome assembling.**
(XLS)

File S1 **Figure S1.** Volcano plot displaying the −log_10_ of the *P* values from Kal's statistical test in terms of the log_2_ fold change for nauplius I–II/copepodid, copepodid/chalimus and female/male of *C. rogercresseyi*. The selected genes have significantly different expression values (P≤10^−16^–P≤10^−5^). Dots, triangles and squares represent individual ESTs from larvae stages and adult salmon lice, respectively. Annotated and unannotated sequences according BLAST analysis as filled and empty spots were denoted. **Figure S2.** Number of contigs annotated and unannotated showing up/down regulation for nauplius I–II/copepodid, copepodid/chalimus and female/male of *C. rogercresseyi*. **Figure S3.** Principal component analysis from six *Caligus rogercresseyi* development stages – nauplius I, nauplius II, copepodid, chalimus and female and male adults. **Figure S4.** Relative expression levels of acetoacetyl-CoA synthetase gene from six developmental stage of *Caligus rogercresseyi*. Each bar represents the mean of expression levels (± SD). **Figure S5.** Relative expression level of flotillin and allatostatin precursor protein from six developmental stage of *Caligus rogercresseyi*. Each bar represents the mean of expression levels (± SD). **Figure S6.** Relative expression level of tropomyosin and putative cuticle protein from six developmental stage of *Caligus rogercresseyi*. Each bar represents the mean of expression levels (± SD). **Figure S7.** Relative expression levels of vitellogenin 1 and 2 gene from six developmental stage of *Caligus rogercresseyi*. Each bar represents the mean of expression levels (± SD). **Figure S8.** Relative expression levels of argonaute 1 isoform C and Vasa gene from six developmental stage of *Caligus rogercresseyi*. Each bar represents the mean of expression levels (± SD). **Figure S9.** Correlation analysis between transformed expression values obtained by qPCR and in silico analysis from six developmental stage of *Caligus rogercresseyi*.
(DOCX)

File S2 **Table S3.** Relevant annotated genes identified by clustering analysis between Nauplius I–II and Copepodid stages of *C. rogercresseyi* transcriptome. **Table S4.** Relevant annotated genes identified by clustering analysis between Copepodid and Chalimus stages of C. rogercresseyi transcriptome. **Table S5.** Relevant annotated genes identified by clustering analysis between Female and Male stages of C. rogercresseyi transcriptome.
(DOCX)

File S3 **Table S6.** Hypothetical proteins and unannotated contigs Up/down-regulated in Copepodid/Nauplius I–II groups. **Table S7.** Hypothetical proteins and unannotated contigs Up/down-regulated in Chalimus/Copepodid groups. **Table S8.** Hypothetical proteins and unannotated contigs Unannotated contigs Up/down-regulated in Male/Female groups.
(DOCX)
