# Supplementary material for: RNA-Seq Analysis Using De Novo Transcriptome Assembly as a Reference for the Salmon Louse Caligus rogercresseyi
Source: PLoS One. 2014 Apr 1;9(4):e92239. doi: 10.1371/journal.pone.0092239 (PMC3972170; doi:10.1371/journal.pone.0092239)
Supplement: Table S1 — Primer list for qPCR validated in C. rogercresseyi genes. (DOCX) [file pone.0092239.s001.docx]

**Table S1.** Primer list for *C. rogercresseyi* genes validated by qPCR

| **Primer Name** | **Primer Sequence** | **Ta (ºC)** |
| --- | --- | --- |
| contig25754_F2 | CACCTCCGAGTTTATTCGCT | 58 |
| contig25754_R2 | AGGACTTGAGTTGGCTTTCC |  |
| contig3464_F2 | CGCAAGGACGACAATTTCAA | 58 |
| contig3464_R2 | TAATTGATGGCCTTCCGTGG |  |
| contig1263_F1 | ATGTCTCTTCCACCCAGGAT | 58 |
| contig1263_R1 | CCGCATCAATAGCTTTTCCG |  |
| contig56379_F1 | AATCCAAGTCCATCAGCGAC | 58 |
| contig56379_R1 | CTCCAACATCAACTCTGCCA |  |
| contig61222_F2 | TACGTCCTTCAGCTTAGCCT | 58 |
| contig61222_R2 | GAAACCACGAAAATGGCCTC |  |
| contig21663_F2 | GGCTCAGACAAGAAGCATGA | 58 |
| contig21663_R2 | TCCTCTTTCCCAGTCCGAAT |  |
| contig5709_F2 | TAACCAGAGCAAGGAGTCCA | 58 |
| contig5709_R2 | TGCTCTTGAGGTGGGAGTAA |  |
| contig30958_F1 | CCATTTCCGTCACTGGGATT | 58 |
| contig30958_R1 | TTTGATGACCCTCAAGCGTT |  |
| contig23903_F1 | CATGTTACCGGACACTTCCA | 58 |
| contig23903_R1 | TTAATGTGTCTCTCGGGGGT |  |
| Cr_b-tubulin_2F | TTTGTTGTGTGAGCTCTGGG | 60 |
| Cr_b-tubulin_2R | GCTGATCTCCGAAAACTTGC |  |
|  |  |  |
