# Supplementary material for: RNA-Seq Analysis Using De Novo Transcriptome Assembly as a Reference for the Salmon Louse Caligus rogercresseyi
Source: PLoS One. 2014 Apr 1;9(4):e92239. doi: 10.1371/journal.pone.0092239 (PMC3972170; doi:10.1371/journal.pone.0092239)
Supplement: File S1 — Figure S1. Volcano plot displaying the −log10 of the P values from Kal's statistical test in terms of the log2 fold change for nauplius I–II/copepodid, copepodid/chalimus and female/male of C. rogercresseyi. The selected genes have significantly different expression values (P≤10−16–P≤10−5). Dots, triangles and squares represent individual ESTs from larvae stages and adult salmon lice, respectively. Annotated and unannotated sequences according BLAST analysis as filled and empty spots were denoted. Figure S2. Number of contigs annotated and unannotated showing up/down regulation for nauplius I–II/copepodid, copepodid/chalimus and female/male of C. rogercresseyi. Figure S3. Principal component analysis from six Caligus rogercresseyi development stages – nauplius I, nauplius II, copepodid, chalimus and female and male adults. Figure S4. Relative expression levels of acetoacetyl-CoA synthetase gene from six developmental stage of Caligus rogercresseyi. Each bar represents the mean of expression levels (± SD). Figure S5. Relative expression level of flotillin and allatostatin precursor protein from six developmental stage of Caligus rogercresseyi. Each bar represents the mean of expression levels (± SD). Figure S6. Relative expression level of tropomyosin and putative cuticle protein from six developmental stage of Caligus rogercresseyi. Each bar represents the mean of expression levels (± SD). Figure S7. Relative expression levels of vitellogenin 1 and 2 gene from six developmental stage of Caligus rogercresseyi. Each bar represents the mean of expression levels (± SD). Figure S8. Relative expression levels of argonaute 1 isoform C and Vasa gene from six developmental stage of Caligus rogercresseyi. Each bar represents the mean of expression levels (± SD). Figure S9. Correlation analysis between transformed expression values obtained by qPCR and in silico analysis from six developmental stage of Caligus rogercresseyi. (DOCX) [file pone.0092239.s003.docx]

Figure S1. Volcano plot displaying the −log_10_ of the *P* values from Kal’s statistical test in terms of the log_2_ fold change for nauplius I-II/copepodid, copepodid/chalimus and female/male of *C. rogercresseyi*. The selected genes have significantly different expression values (P≤10^−16^–P≤10^−5^). Dots, triangles and squares represent individual ESTs from larvae stages and adult salmon lice, respectively. Annotated and unannotated sequences according BLAST analysis as filled and empty spots were denoted.

Figure S2. Number of contigs annotated and unannotated showing up/down regulation for nauplius I-II/copepodid, copepodid/chalimus and female/male of *C. rogercresseyi*.


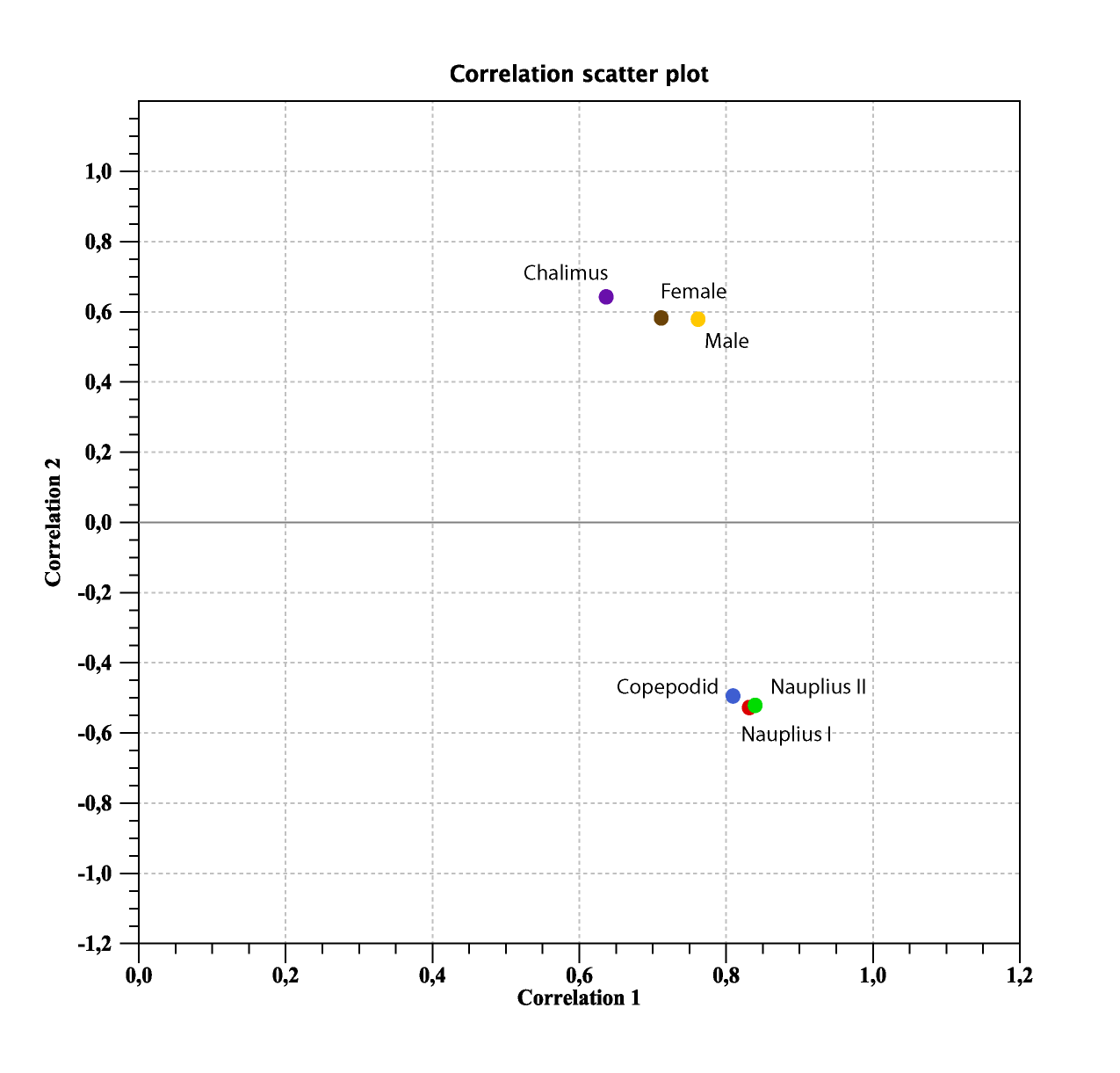


Figure S3. Principal component analysis from six *Caligus rogercresseyi* development stages – nauplius I, nauplius II, copepodid, chalimus and female and male adults.

Figure S4. Relative expression levels of acetoacetyl-CoA synthetase gene from six developmental stage of *Caligus rogercresseyi*. Each bar represents the mean of expression levels (± SD).

Figure S5. Relative expression level of flotillin and allatostatin precursor protein from six developmental stage of *Caligus rogercresseyi*. Each bar represents the mean of expression levels (± SD).

Figure S6. Relative expression level of tropomyosin and putative cuticle protein from six developmental stage of *Caligus rogercresseyi*. Each bar represents the mean of expression levels (± SD).

Figure S7. Relative expression levels of vitellogenin 1 and 2 gene from six developmental stage of *Caligus rogercresseyi*. Each bar represents the mean of expression levels (± SD).

Figure S8. Relative expression levels of argonaute 1 isoform C and Vasa gene from six developmental stage of *Caligus rogercresseyi*. Each bar represents the mean of expression levels (± SD).

Figure S9. Correlation analysis between transformed expression values obtained by qPCR and in silico analysis from six developmental stage of *Caligus rogercresseyi*.
