# Supplementary material for: RNA-Seq Analysis Using De Novo Transcriptome Assembly as a Reference for the Salmon Louse Caligus rogercresseyi
Source: PLoS One. 2014 Apr 1;9(4):e92239. doi: 10.1371/journal.pone.0092239 (PMC3972170; doi:10.1371/journal.pone.0092239)
Supplement: File S2 — Table S3. Relevant annotated genes identified by clustering analysis between Nauplius I–II and Copepodid stages of C. rogercresseyi transcriptome. Table S4. Relevant annotated genes identified by clustering analysis between Copepodid and Chalimus stages of C. rogercresseyi transcriptome. Table S5. Relevant annotated genes identified by clustering analysis between Female and Male stages of C. rogercresseyi transcriptome. (DOCX) [file pone.0092239.s004.docx]

**Table S3. Relevant annotated genes identified by clustering analysis between Nauplius I-II and Copepodid stages of *C. rogercresseyi* transcriptome**

| **Accession no.** | **Lowest E-value** | | **Annotation** | **Fold Change (log2)** | **Redundancy** |
| --- | --- | --- | --- | --- | --- |
| ***Cluster1*** | |  |  |  |  |
| ACO12723 | | 3,37086E-86 | 40S ribosomal protein S3a [Lepeophtheirus salmonis]. | -1,5 | 2 |
| ACO11694 | | 9,59805E-83 | 60S ribosomal protein L13A [Caligus rogercresseyi]. | -1,4 | 3 |
| ACO12488 | | 2,24467E-39 | ADP,ATP carrier protein 3 [Lepeophtheirus salmonis]. | -1,4 | 1 |
| ACO11152 | | 3,34997E-63 | Arginine kinase [Caligus rogercresseyi]. | -1,4 | 1 |
| AEL23055 | | 1,13219E-13 | CHK1 checkpoint-like protein, partial [Cherax quadricarinatus]. | -1,4 | 2 |
| ADM67905 | | 2,44418E-27 | Cytochrome b (mitochondrion) [Caligus clemensi]. | -1,8 | 3 |
| ADM67896 | | 8,7225E-126 | Cytochrome c oxidase subunit II (mitochondrion) [Caligus rogercresseyi]. | -1,3 | 4 |
| ABU41064 | | 5,57464E-28 | Elongation factor 1-alpha [Lepeophtheirus salmonis]. | -1,5 | 1 |
| ADD24273 | | 8,58929E-35 | FK506-binding protein 2 [Lepeophtheirus salmonis]. | -1,4 | 1 |
| ACO12340 | | 2,54723E-39 | Fructose-bisphosphate aldolase [Lepeophtheirus salmonis]. | -1,4 | 1 |
| ACO12532 | | 8,17292E-55 | Gamma-interferon-inducible lysosomal thiol reductase [Lepeophtheirus salmonis]. | -1,6 | 1 |
| ADM67895 | | 5,53408E-67 | NADH dehydrogenase subunit 5 (mitochondrion) [Caligus rogercresseyi]. | -1,4 | 2 |
| P62251 | | 2,56888E-67 | RS16_AEDAE 40S ribosomal protein S16 OS=Aedes aegypti | -1,5 | 1 |
| ACO11567 | | 1,86976E-88 | 60S ribosomal protein L17 [Caligus rogercresseyi]. | -1,6 | 1 |
| ABU41079 | | 4,9744E-116 | Tryptophan 5-monooxygenase activation protein [Lepeophtheirus salmonis]. | -1,4 | 1 |
| ***Cluster2*** | |  |  |  |  |
| ABU41136 | | 2,71837E-14 | Vitellogenin-like protein [Lepeophtheirus salmonis]. | -10,8 | 1 |
| ABU41135 | | 8,03483E-37 | Vitellogenin 2 [Lepeophtheirus salmonis]. | -7,5 | 1 |
| ACO10773 | | 1,59612E-23 | Troponin C, isoform 1 [Caligus rogercresseyi]. | 1,7 | 1 |
| ACO10496 | | 1,37621E-20 | Sarcoplasmic calcium-binding protein, beta chain [Caligus rogercresseyi]. | 2,2 | 1 |
| ACO14617 | | 6,96336E-68 | Ras-related protein Rab-3 [Caligus clemensi]. | 2,0 | 1 |
| ABU41130 | | 0,00938142 | Putative SPT transcription factor family member, partial [Lepeophtheirus salmonis]. | 2,1 | 1 |
| ABU41117 | | 1,64416E-19 | Putative metalloproteinase [Lepeophtheirus salmonis]. | -1,5 | 1 |
| ABU41082 | | 5,01776E-53 | Putative cuticle protein [Lepeophtheirus salmonis]. | 2,5 | 6 |
| ADD24043 | | 1,00058E-10 | Protein ZK643.6 [Lepeophtheirus salmonis]. | -1,9 | 2 |
| ABU41061 | | 8,32818E-55 | Protein disulfide-isomerase 2 precursor, partial [Lepeophtheirus salmonis]. | 3,1 | 1 |
| ADM67895 | | 3,3348E-121 | NADH dehydrogenase subunit 5 (mitochondrion) [Caligus rogercresseyi]. | -10,3 | 3 |
| ACO15400 | | 2,76444E-27 | Myosin light chain alkali [Caligus clemensi]. | 9,0 | 2 |
| ACO15229 | | 1,52253E-49 | Mite group 2 allergen Pso o 2 precursor [Caligus clemensi]. | 1,9 | 1 |
| ABU41019 | | 6,85936E-09 | Metalloproteinase, partial [Lepeophtheirus salmonis]. | 2,2 | 6 |
| ADD38666 | | 0,0365354 | Matrix metalloproteinase-9 [Lepeophtheirus salmonis]. | -1,9 | 1 |
| Q5F3N5 | | 1,25135E-07 | KLH14_CHICK Kelch-like protein 14 OS=Gallus gallus | 5,8 | 1 |
| ABU41084 | | 1,00058E-73 | Hypothetical protein [Lepeophtheirus salmonis]. | -2,8 | 27 |
| AAP49384 | | 8,44709E-11 | Glutamate dehydrogenase [Tigriopus californicus]. | 2,0 | 1 |
| ADD38289 | | 7,89573E-91 | Gamma-crystallin A [Lepeophtheirus salmonis]. | 12,9 | 1 |
| ACO12937 | | 8,90889E-05 | Flexible cuticle protein 12 precursor [Lepeophtheirus salmonis]. | -2,5 | 1 |
| ACO10277 | | 2,57261E-23 | Eukaryotic translation initiation factor 5A [Caligus rogercresseyi]. | 3,8 | 1 |
| AFI39731 | | 6,21663E-90 | enolase, partial [Daphnia parvula]. | 2,5 | 1 |
| ACO11766 | | 4,21268E-80 | E-selectin precursor [Lepeophtheirus salmonis]. | 2,6 | 1 |
| ACO14561 | | 2,32173E-30 | Cuticle protein 18.6, isoform B [Caligus clemensi]. | -1,6 | 3 |
| ACO14743 | | 2,2708E-123 | Collagenase precursor [Caligus clemensi]. | 2,7 | 1 |
| ACO10881 | | 6,5465E-39 | Carbonic anhydrase 1 [Caligus rogercresseyi]. | -2,1 | 1 |
| ACO11425 | | 7,86399E-11 | ATP synthase subunit d, mitochondrial [Caligus rogercresseyi]. | -6,0 | 1 |
| ACO10612 | | 2,8566E-103 | Ascorbate peroxidase precursor [Caligus rogercresseyi]. | -2,6 | 1 |
| A2BH40 | | 1,19194E-19 | AT-rich interactive domain-containing protein 1A OS=Mus musculus | 2,0 | 1 |
| ACO11785 | | 6,53548E-25 | Arginine kinase [Lepeophtheirus salmonis]. | 8,0 | 1 |
| ACO11157 | | 0 | Alaserpin precursor [Caligus rogercresseyi]. | -14,6 | 1 |
| ABU41089 | | 4,23941E-88 | actin [Lepeophtheirus salmonis]. | -2,5 | 3 |
| EFX73813 | | 3,84426E-15 | ABC protein, subfamily ABCF [Daphnia pulex]. | 1,7 | 1 |
| ACO10287 | | 2,33422E-38 | 14-3-3 protein gamma-B [Caligus rogercresseyi]. | 2,1 | 1 |
| ***Cluster3*** | |  |  |  |  |
| ACO15258 | | 6,25281E-50 | 60S ribosomal protein L18 [Caligus clemensi]. | -1,6 | 7 |
| AAT98704 | | 1,2963E-117 | Cytochrome b (mitochondrion) [Lepeophtheirus salmonis]. | -1,8 | 1 |
| ABU41064 | | 4,03275E-28 | Elongation factor 1-alpha [Lepeophtheirus salmonis]. | -1,9 | 2 |
| EFX59970 | | 3,35805E-15 | Hypothetical protein DAPPUDRAFT_126039 [Daphnia pulex]. | -1,7 | 2 |
| ABU41053 | | 8,624E-106 | Metalloproteinase [Lepeophtheirus salmonis]. | -1,6 | 1 |
| ACG63696 | | 8,95274E-32 | Myosin heavy chain, partial [Gammarus duebeni]. | -2,0 | 1 |
| ADM67895 | | 7,35293E-48 | NADH dehydrogenase subunit 5 (mitochondrion) [Caligus rogercresseyi]. | 4,7 | 1 |
| ACO11691 | | 2,86085E-19 | ORF91 [Caligus rogercresseyi]. | -1,8 | 2 |
| ABU41117 | | 5,22943E-26 | Putative metalloproteinase [Lepeophtheirus salmonis]. | -1,7 | 1 |
| ACO12887 | | 3,30817E-69 | Troponin T [Lepeophtheirus salmonis]. | -1,6 | 1 |
| ***Cluster4*** | |  |  |  |  |
| ABU41136 | | 3,04598E-05 | Vitellogenin-like protein [Lepeophtheirus salmonis]. | 3,8 | 1 |
| ACO11835 | | 6,79775E-27 | Vacuolar ATP synthase 16 kDa proteolipid subunit [Lepeophtheirus salmonis]. | 1,5 | 1 |
| AAB00497 | | 2,5092E-40 | Ubiquitin, partial [Homarus americanus]. | 1,8 | 1 |
| ACO10194 | | 6,3852E-118 | Trypsin epsilon precursor [Caligus rogercresseyi]. | 1,7 | 1 |
| ACO11077 | | 1,05829E-75 | Troponin C, isoform 1 [Caligus rogercresseyi]. | 3,3 | 2 |
| ACO12903 | | 2,05405E-23 | Triosephosphate isomerase [Lepeophtheirus salmonis]. | 2,0 | 1 |
| ACO10911 | | 3,3866E-23 | Thioredoxin-2 [Caligus rogercresseyi]. | 1,9 | 1 |
| P28551 | | 5,44961E-39 | TBB3_SOYBN Tubulin beta chain | 1,6 | 2 |
| ACO11270 | | 1,0795E-120 | Superoxide dismutase [Caligus rogercresseyi]. | 2,6 | 1 |
| AGN29634 | | 4,84096E-38 | SPT transcription factor family member [Acartia pacifica]. | 2,1 | 1 |
| Q9VE46 | | 0 | High-affinity choline transporter 1 OS=Drosophila melanogaster | 2,1 | 1 |
| ABU41124 | | 8,52173E-35 | Putative cuticle protein, partial [Lepeophtheirus salmonis]. | -3,4 | 5 |
| ABU41078 | | 2,2983E-55 | Putative cuticle protein [Lepeophtheirus salmonis]. | -31,4 | 14 |
| EFX67330 | | 1,72613E-10 | Putative CCCTC-binding factor protein [Daphnia pulex]. | 1,9 | 1 |
| CBW54879 | | 6,26309E-32 | Putative actin, partial [Cancer pagurus]. | 2,6 | 2 |
| ACO12136 | | 3,19226E-32 | Pupal cuticle protein 20 precursor [Lepeophtheirus salmonis]. | 2,1 | 3 |
| AFP92126 | | 6,08866E-06 | plasmolipin isoform 1 [Penaeus monodon]. | 1,5 | 1 |
| ACO10262 | | 1,24137E-45 | Phosphoglycerate mutase 2 [Caligus rogercresseyi]. | 1,5 | 1 |
| ACO12093 | | 5,1421E-127 | PDZ and LIM domain protein 1 [Lepeophtheirus salmonis]. | 2,1 | 1 |
| P34329 | | 3,37532E-09 | Probable protein disulfide-isomerase A4 OS=Caenorhabditis elegans | 2,3 | 1 |
| ADM67895 | | 2,7007E-123 | NADH dehydrogenase subunit 5 (mitochondrion) [Caligus rogercresseyi]. | 2,7 | 3 |
| AGF90965 | | 0 | Na+/K+-ATPase [Portunus trituberculatus]. | 1,4 | 2 |
| ACO10369 | | 3,2469E-58 | Muscle LIM protein Mlp84B [Caligus rogercresseyi]. | 1,6 | 2 |
| ABU41019 | | 5,09513E-39 | Metalloproteinase, partial [Lepeophtheirus salmonis]. | 3,2 | 2 |
| ABU41053 | | 1,37039E-48 | Metalloproteinase [Lepeophtheirus salmonis]. | 9,6 | 5 |
| EFX85390 | | 1,89993E-05 | Hypothetical protein DAPPUDRAFT_99002 [Daphnia pulex]. | 2,3 | 29 |
| ABU41084 | | 0 | Hypothetical protein [Lepeophtheirus salmonis]. | -79,4 | 32 |
| ACO10888 | | 1,93932E-11 | Heat shock protein beta-1 [Caligus rogercresseyi]. | 1,6 | 1 |
| ADD38896 | | 5,01038E-62 | Four and a half LIM domains protein 2 [Lepeophtheirus salmonis]. | 1,9 | 3 |
| ACO12937 | | 6,76375E-47 | Flexible cuticle protein 12 precursor [Lepeophtheirus salmonis]. | 1,5 | 1 |
| ABD96095 | | 3,3315E-106 | Elongation factor 1-alpha, partial [Rhincalanus nasutus]. | -2,8 | 1 |
| ACO11336 | | 1,59208E-10 | DnaJ homolog subfamily A member 1 [Caligus rogercresseyi]. | 1,9 | 1 |
| ACO10726 | | 1,38058E-21 | Disulfide-isomerase A3 precursor [Caligus rogercresseyi]. | 2,2 | 1 |
| ADD37964 | | 6,62572E-74 | Cuticle protein 7 [Lepeophtheirus salmonis]. | 1,9 | 3 |
| ADD38018 | | 7,47169E-43 | Cuticle protein 6 [Lepeophtheirus salmonis]. | 1,5 | 4 |
| AFA46584 | | 5,19852E-31 | Collagen alpha-1 IV chain precursor-like protein, partial [Armillifer agkistrodontis]. | 2,8 | 1 |
| ACO10881 | | 1,13531E-53 | Carbonic anhydrase 1 [Caligus rogercresseyi]. | -1,6 | 2 |
| ABU41123 | | 1,16544E-23 | BCS-1-like protein [Lepeophtheirus salmonis]. | -1,6 | 2 |
| ADM67892 | | 8,373E-108 | ATP synthase F0 subunit 6 (mitochondrion) [Caligus rogercresseyi]. | 3,0 | 6 |
| ACO11785 | | 7,2939E-153 | Arginine kinase [Lepeophtheirus salmonis]. | 2,0 | 3 |
| BAF64528 | | 3,93537E-11 | Allatostatin precursor protein [Panulirus interruptus]. | 1,6 | 1 |
| ACO12057 | | 2,60019E-16 | 60S ribosomal protein L14 [Lepeophtheirus salmonis]. | 1,7 | 1 |
| ACO10247 | | 8,96346E-20 | 40S ribosomal protein S9 [Caligus rogercresseyi]. | 2,2 | 2 |

**Table S4. Relevant annotated genes identified by clustering analysis between Copepodid and Chalimus stages of *C. rogercresseyi* transcriptome**

| **Accession no.** | **Lowest E-value** | **Annotation** | **Fold Change (log2)** | **Redundancy** |
| --- | --- | --- | --- | --- |
| ***Cluster1*** |  |  |  |  |
| EFX78468 | 0 | ABC protein, subfamily ABCH [Daphnia pulex]. | -1,35 | 3 |
| ABU41093 | 1,63215E-32 | BCS-1 protein, partial [Lepeophtheirus salmonis]. | 2,23 | 4 |
| Q17IE8 | 0 | CDK8_AEDAE Cyclin-dependent kinase 8 [Aedes aegypti]. | -1,33 | 2 |
| ADD24462 | 6,33196E-11 | Cerebellin-3 [Lepeophtheirus salmonis]. | -1,37 | 1 |
| ACO11940 | 5,7274E-126 | Clarin-3 [Lepeophtheirus salmonis]. | -1,99 | 1 |
| ACO12785 | 1,98703E-49 | Cuticle protein CP14.6 precursor [Lepeophtheirus salmonis]. | -2,53 | 3 |
| ACO15102 | 5,48001E-34 | High mobility group protein B2 [Caligus clemensi]. | -1,30 | 1 |
| ABU41084 | 2,3392E-106 | Hypothetical protein [Lepeophtheirus salmonis]. | -2,19 | 17 |
| EFX86494 | 0 | Hypothetical protein DAPPUDRAFT_192774 [Daphnia pulex]. | -1,92 | 10 |
| ACO13109 | 1,14068E-30 | Neuronal acetylcholine receptor subunit alpha-3 precursor [Lepeophtheirus salmonis]. | -1,65 | 4 |
| ABU41124 | 2,36173E-34 | Putative cuticle protein, partial [Lepeophtheirus salmonis]. | -2,74 | 7 |
| Q9VE46 | 0 | SC5A7_DROME High-affinity choline transporter 1 [Drosophila melanogaster]. | 2,00 | 1 |
| Q9XZC8 | 0 | SEM2A_SCHGR Semaphorin-2A [Schistocerca gregaria]. | -1,94 | 1 |
| ACO11818 | 1,14263E-95 | Torso-like protein precursor [Lepeophtheirus salmonis]. | -2,88 | 2 |
| ACO13169 | 2,90774E-50 | Tropomyosin [Lepeophtheirus salmonis]. | -2,47 | 2 |
| ***Cluster2*** |  |  |  |  |
| ABU41089 | 2,2311E-163 | Actin [Lepeophtheirus salmonis]. | -1,15 | 16 |
| ACO15100 | 7,13066E-93 | Acetylcholine receptor subunit beta-like 1 precursor [Caligus clemensi]. | -2,47 | 1 |
| ADD38615 | 9,3172E-159 | Adenosylhomocysteinase [Lepeophtheirus salmonis]. | 22,96 | 3 |
| ACO15391 | 0 | Agglutinin isolectin 1 precursor [Caligus clemensi]. | -1,23 | 2 |
| ADK39312 | 1,58153E-89 | Akirin 2, partial [Caligus rogercresseyi]. | -1,12 | 1 |
| EFX86288 | 0 | Akt1-like protein [Daphnia pulex]. | -1,08 | 1 |
| ACO11785 | 7,2939E-153 | Arginine kinase [Lepeophtheirus salmonis]. | -7,77 | 3 |
| AEI83215 | 4,546E-150 | Arsenite-resistance protein 2-like protein [Litopenaeus vannamei]. | -1,13 | 1 |
| ADD38187 | 5,2304E-162 | Ascorbate peroxidase [Lepeophtheirus salmonis]. | 7,39 | 1 |
| ACO15551 | 2,325E-121 | Calcium-binding protein p22 [Caligus clemensi]. | -1,12 | 3 |
| ACO14561 | 3,85912E-34 | Cuticle protein 18.6, isoform B [Caligus clemensi]. | -1,68 | 10 |
| ACO12089 | 1,46512E-54 | Cuticle protein 19 [Lepeophtheirus salmonis]. | -56,41 | 8 |
| ADD38018 | 7,47169E-43 | Cuticle protein 6 [Lepeophtheirus salmonis]. | -1,23 | 3 |
| ACO12414 | 1,00186E-22 | Cuticle protein 7 [Lepeophtheirus salmonis]. | -17,07 | 11 |
| ACO11766 | 1,25313E-98 | E-selectin precursor [Lepeophtheirus salmonis]. | -10,39 | 2 |
| ACO10536 | 7,1077E-99 | Ferritin subunit precursor [Caligus rogercresseyi]. | -1,08 | 3 |
| ADD38355 | 0 | Guanine nucleotide-binding protein subunit beta-1 [Lepeophtheirus salmonis]. | -1,40 | 2 |
| ABU41084 | 2,7575E-175 | Hypothetical protein [Lepeophtheirus salmonis]. | -12,00 | 20 |
| ACO11064 | 7,2691E-139 | Lethal2essential for life [Caligus rogercresseyi]. | -1,08 | 1 |
| ACO15084 | 9,70384E-74 | Myosin light chain alkali [Caligus clemensi]. | -1,05 | 4 |
| ACO13186 | 3,88071E-45 | Myosin light chain alkali [Lepeophtheirus salmonis]. | -1,12 | 3 |
| ACO10487 | 2,5748E-144 | Neuronal acetylcholine receptor subunit alpha-3 precursor [Caligus rogercresseyi]. | -1,56 | 3 |
| ABU41068 | 1,82904E-45 | Putative cuticle protein [Lepeophtheirus salmonis]. | -1,40 | 26 |
| ACO11121 | 0 | Septin-2 [Caligus rogercresseyi]. | -1,08 | 7 |
| ADD24466 | 1,7169E-106 | Ubiquitin [Lepeophtheirus salmonis]. | -1,33 | 4 |
| ***Cluster3*** |  |  |  |  |
| ADD38111 | 8,44641E-50 | Beta-crystallin A1 [Lepeophtheirus salmonis]. | 6,96 | 3 |
| ACO15299 | 3,70785E-41 | Collagenase precursor [Caligus clemensi]. | 7,95 | 3 |
| Q07981 | 8,87667E-07 | FAR1_HELAS FMRFamide-related peptides type [Helix aspersa] | 2,18 | 2 |
| ADD38289 | 6,09262E-44 | Gamma-crystallin A [Lepeophtheirus salmonis]. | 13,60 | 5 |
| ABU41105 | 2,31346E-22 | Hypothetical protein [Lepeophtheirus salmonis]. | 5,17 | 4 |
| ACS36133 | 3,97062E-06 | PH glutathione peroxidase, partial [Tigriopus japonicus]. | 5,79 | 1 |
| ACO10262 | 4,47137E-63 | Phosphoglycerate mutase 2 [Caligus rogercresseyi]. | 2,54 | 1 |
| ABU41074 | 4,03485E-89 | Phospholipid-hydroperoxide glutathione peroxidase [Lepeophtheirus salmonis]. | 2,33 | 1 |
| ADD38689 | 8,59182E-71 | Serine protease K12H4.7 [Lepeophtheirus salmonis]. | 4,83 | 5 |
| ACO11532 | 2,695E-142 | Trypsin-1 [Caligus rogercresseyi]. | 14,76 | 10 |
| AFD54569 | 0 | Vasa [Lepeophtheirus salmonis]. | 2,49 | 3 |
| ***Cluster4*** |  |  |  |  |
| ACO10992 | 1,3804E-147 | Carbonic anhydrase 6 [Caligus rogercresseyi]. | -13,76 | 7 |
| ACO10795 | 2,5109E-62 | Carbonic anhydrase 3 [Caligus rogercresseyi]. | -13,28 | 1 |
| ACO10242 | 1,07981E-65 | Carboxypeptidase B [Caligus rogercresseyi]. | -5,07 | 2 |
| ADM53739 | 6,8029E-129 | Cathepsin L2 precursor [Lepeophtheirus salmonis]. | -16,70 | 2 |
| ABU41053 | 4,68291E-43 | Metalloproteinase [Lepeophtheirus salmonis]. | -2,72 | 18 |
| ADF87945 | 2,80153E-40 | Peroxinectin [Eriocheir sinensis]. | -2,69 | 2 |
| CAO00434 | 1,6052E-120 | Unnamed protein product [Lepeophtheirus salmonis]. | -4,89 | 30 |
| ABU41134 | 1,4716E-154 | Vitellogenin 1 [Lepeophtheirus salmonis]. | -3,67 | 15 |
| ABU41135 | 3,295E-137 | Vitellogenin 2 [Lepeophtheirus salmonis]. | -5,03 | 14 |
| ABU41136 | 0 | Vitellogenin-like protein [Lepeophtheirus salmonis]. | -6,87 | 31 |
| ***Cluster5*** |  |  |  |  |
| ACO11045 | 1,94506E-46 | 40S ribosomal protein S18 [Caligus rogercresseyi]. | 1,17 | 2 |
| ADD38360 | 2,02199E-65 | 40S ribosomal protein S20 [Lepeophtheirus salmonis]. | 1,28 | 3 |
| ACO12708 | 1,9321E-130 | 40S ribosomal protein S4 [Lepeophtheirus salmonis]. | 1,42 | 7 |
| ACO10370 | 1,17001E-66 | 60S ribosomal protein L10a [Caligus rogercresseyi]. | 1,16 | 3 |
| ACO11694 | 3,75505E-89 | 60S ribosomal protein L13A [Caligus rogercresseyi]. | 1,19 | 5 |
| ACO15652 | 4,89475E-46 | 60S ribosomal protein L21 [Caligus clemensi]. | 1,19 | 5 |
| ACO10367 | 2,45508E-54 | 60S ribosomal protein L35 [Caligus rogercresseyi]. | 1,81 | 6 |
| ADD38662 | 1,31057E-58 | 60S ribosomal protein L37a [Lepeophtheirus salmonis]. | 1,19 | 2 |
| ACO10217 | 3,7584E-24 | 60S ribosomal protein L39 [Caligus rogercresseyi]. | 1,14 | 2 |
| ACO11152 | 4,1416E-178 | Arginine kinase [Caligus rogercresseyi]. | 1,11 | 19 |
| ACO15246 | 2,0467E-144 | Aspartate aminotransferase, cytoplasmic [Caligus clemensi]. | 1,37 | 2 |
| ACO14840 | 0 | Carboxypeptidase B [Caligus clemensi]. | 1,91 | 3 |
| ACO11291 | 4,70221E-94 | Cellular retinoic acid-binding protein 2 [Caligus rogercresseyi]. | 1,11 | 3 |
| ACO11095 | 1,01766E-39 | Charged multivesicular body protein 1a [Caligus rogercresseyi]. | 1,08 | 2 |
| ABU41064 | 4,5555E-114 | Elongation factor 1-alpha [Lepeophtheirus salmonis]. | 2,17 | 10 |
| ABU41067 | 2,3212E-177 | enolase [Lepeophtheirus salmonis]. | 1,41 | 11 |
| ACO12340 | 6,9407E-114 | Fructose-bisphosphate aldolase [Lepeophtheirus salmonis]. | 1,41 | 16 |
| ABI95854 | 1,00428E-21 | GABA-alpha subunit, partial [Lepeophtheirus salmonis]. | 1,07 | 2 |
| ABU41037 | 1,3157E-129 | Glyceraldehyde-3-phosphate dehydrogenase [Lepeophtheirus salmonis]. | 1,35 | 16 |
| ACA03523 | 0 | Heat shock protein 70 precursor [Tigriopus japonicus]. | 1,06 | 2 |
| ACA03524 | 7,17623E-68 | Heat shock protein 90 [Tigriopus japonicus]. | 1,03 | 2 |
| ACO11619 | 7,5398E-141 | Sequestosome-1 [Caligus rogercresseyi]. | 1,07 | 5 |
| ACO11270 | 1,0795E-120 | Superoxide dismutase [Caligus rogercresseyi]. | 1,35 | 2 |
| ACO10221 | 7,1311E-159 | Transmembrane protein 49 [Caligus rogercresseyi]. | 1,18 | 3 |
| ABU41102 | 3,0039E-81 | Zeelin1-like protein [Lepeophtheirus salmonis]. | 1,16 | 2 |

**Table S5. Relevant annotated genes identified by clustering analysis between Female and Male stages of *C. rogercresseyi* transcriptome.**

| **Accession no.** | **Lowest E-value** | **Annotation** | **Fold Change (log2)** | **Redundancy** |
| --- | --- | --- | --- | --- |
| ***Cluster1*** |  |  |  |  |
| ABU41123 | 1,02275E-22 | BCS-1-like protein [Lepeophtheirus salmonis]. | 10,14 | 2 |
| ACO11422 | 1,479E-156 | Bleomycin hydrolase [Caligus rogercresseyi]. | -1,93 | 2 |
| ACO11031 | 3,94377E-14 | Carbonic anhydrase 6 precursor [Caligus rogercresseyi]. | -8,97 | 2 |
| ACO10457 | 2,3815E-135 | Carboxypeptidase B [Caligus rogercresseyi]. | -2,12 | 3 |
| ADD24515 | 6,36529E-85 | Cuticle protein 6 [Lepeophtheirus salmonis]. | 8,47 | 9 |
| ABU41084 | 0 | Hypothetical protein [Lepeophtheirus salmonis]. | 7,07 | 10 |
| ACO12924 | 9,44343E-56 | Myosin light chain alkali [Lepeophtheirus salmonis]. | -18,60 | 2 |
| ABU41082 | 1,09423E-62 | Putative cuticle protein [Lepeophtheirus salmonis]. | -12,48 | 14 |
| ACO12080 | 0 | Tubulin alpha-1C chain [Lepeophtheirus salmonis]. | -2,00 |  |
| ***Cluster2*** |  |  |  |  |
| ACO11607 | 3,39418E-34 | 40S ribosomal protein S15Aa [Caligus rogercresseyi]. | -3,81 | 2 |
| ACO11190 | 6,14371E-65 | 40S ribosomal protein S2 [Caligus rogercresseyi]. | -1,28 | 3 |
| ACO10425 | 6,0754E-101 | 60S ribosomal protein L10 [Caligus rogercresseyi]. | -1,29 | 3 |
| ACO11567 | 5,3672E-125 | 60S ribosomal protein L17 [Caligus rogercresseyi]. | -4,32 | 3 |
| ACO11463 | 1,63445E-87 | 60S ribosomal protein L3 [Caligus rogercresseyi]. | -1,22 | 4 |
| ABU41033 | 6,111E-105 | 60S ribosomal protein L5 [Lepeophtheirus salmonis]. | -1,15 | 4 |
| ACO14694 | 4,7611E-143 | 60S ribosomal protein L7 [Caligus clemensi]. | -1,26 | 3 |
| EFX88800 | 0 | ABC protein, subfamily ABCF [Daphnia pulex]. | -1,13 | 2 |
| ACO11880 | 9,1455E-147 | Adenosylhomocysteinase [Lepeophtheirus salmonis]. | -7,47 | 2 |
| ACO12521 | 0 | Arginine N-methyltransferase 1 [Lepeophtheirus salmonis]. | -1,52 | 1 |
| ADD38501 | 4,60667E-31 | C-Myc-binding protein [Lepeophtheirus salmonis]. | -1,19 | 1 |
| ACO10992 | 1,86117E-43 | Carbonic anhydrase 6 [Caligus rogercresseyi]. | -3,31 | 1 |
| ACO14903 | 5,4309E-131 | Cathepsin L precursor [Caligus clemensi]. | -6,82 | 1 |
| EFX74207 | 1,7046E-112 | Cct5-prov protein [Daphnia pulex]. | -1,24 | 3 |
| ACO12664 | 2,10693E-42 | Cuticle protein 7 [Lepeophtheirus salmonis]. | -2,72 | 3 |
| ACO11498 | 2,2069E-116 | Deoxynucleoside kinase [Caligus rogercresseyi]. | -4,50 | 2 |
| ACO11821 | 0 | DnaJ homolog subfamily A member 1 [Lepeophtheirus salmonis]. | -1,22 | 3 |
| ABD96092 | 1,19355E-85 | Elongation factor 1-alpha, partial [Eucalanus hyalinus]. | -1,31 | 2 |
| ACO10807 | 3,0485E-140 | ER lumen protein retaining receptor [Caligus rogercresseyi]. | -1,02 | 1 |
| ACO10725 | 0 | Eukaryotic translation initiation factor 3 [Caligus rogercresseyi]. | -1,21 | 8 |
| ADD38289 | 1,19123E-47 | Gamma-crystallin A [Lepeophtheirus salmonis]. | -1,10 | 3 |
| ACM66845 | 4,89706E-24 | Nuclear autoantigenic sperm protein [Penaeus monodon]. | -3,26 | 3 |
| ACO12581 | 5,3124E-150 | Peroxiredoxin-4 [Lepeophtheirus salmonis]. | -1,06 | 2 |
| ACO12282 | 7,0096E-180 | Prohibitin-2 [Lepeophtheirus salmonis]. | -1,18 | 2 |
| ACO12959 | 9,2889E-166 | Proteasome subunit alpha type-4 [Lepeophtheirus salmonis]. | -1,13 | 3 |
| ACO12576 | 8,9198E-50 | Proteasome subunit alpha type-7 [Lepeophtheirus salmonis]. | -1,09 | 3 |
| ACO11652 | 2,7902E-161 | Proteasome subunit beta type-1 precursor [Caligus rogercresseyi]. | -1,11 | 4 |
| ADK94870 | 2,50435E-30 | Vaposin isoform 1 [Penaeus monodon]. | -1,13 | 2 |
| ABU41134 | 2,14834E-45 | Vitellogenin 1 [Lepeophtheirus salmonis]. | -1,42 | 2 |
| ABU41136 | 0 | Vitellogenin-like protein [Lepeophtheirus salmonis]. | -1,24 | 8 |
| ***Cluster3*** |  |  |  |  |
| ACO10992 | 5,1445E-108 | Carbonic anhydrase 6 [Caligus rogercresseyi]. | -2,19 | 2 |
| AEL23055 | 1,36382E-13 | CHK1 checkpoint-like protein, partial [Cherax quadricarinatus]. | -1,66 | 3 |
| P23128 | 4,6012E-106 | Putative ATP-dependent RNA helicase [Drosophila melanogaster] | -2,25 | 2 |
| Q0Q029 | 2,72482E-05 | Putative defense protein 1 [Antheraea mylitta] | 2,25 | 1 |
| Q28IU1 | 9,1818E-66 | Estradiol 17-beta-dehydrogenase [Xenopus tropicalis] | -83,27 | 1 |
| ABU41035 | 4,437E-138 | Hemicentin protein, partial [Lepeophtheirus salmonis]. | 5,37 | 12 |
| ABU41045 | 1,48709E-66 | Hypothetical protein [Lepeophtheirus salmonis]. | 4,14 | 6 |
| ACO10191 | 1,53677E-80 | Mitotic apparatus protein p62 [Caligus rogercresseyi]. | 2,57 | 2 |
| ACM66845 | 1,09674E-19 | Nuclear autoantigenic sperm protein [Penaeus monodon]. | -1,58 | 1 |
| ABU41025 | 1,82754E-52 | Putative cuticle protein [Lepeophtheirus salmonis]. | -1,86 | 6 |
| ACO12796 | 1,8623E-128 | RING finger protein unkempt [Lepeophtheirus salmonis]. | -2,44 | 2 |
| ACO11179 | 1,08795E-26 | Sarcoplasmic calcium-binding protein [Caligus rogercresseyi]. | -2,72 | 1 |
| EFX82785 | 2,09551E-09 | synaptotagmin-like protein 2 variant 1 [Daphnia pulex]. | 21,21 | 1 |
| ACO15408 | 0 | Transmembrane protein nessy [Caligus clemensi]. | -41,77 | 2 |
| ABU41009 | 7,0289E-117 | TSP1-containing protein, partial [Lepeophtheirus salmonis]. | 2,99 | 21 |
| CAO00433 | 2,9727E-128 | Unnamed protein product [Lepeophtheirus salmonis]. | 5,71 | 30 |
| AFD54569 | 0 | Vasa [Lepeophtheirus salmonis]. | -2,02 | 2 |
| ACO10214 | 0 | Venom allergen 5 [Caligus rogercresseyi]. | 1,55 | 1 |
| ABU41134 | 3,9595E-179 | Vitellogenin 1 [Lepeophtheirus salmonis]. | 3,24 | 33 |
| ABU41135 | 3,295E-137 | Vitellogenin 2 [Lepeophtheirus salmonis]. | -7,92 | 62 |
| ABU41136 | 3,0892E-128 | Vitellogenin-like protein [Lepeophtheirus salmonis]. | -1,47 | 10 |
| ***Cluster4*** |  |  |  |  |
| ABU41089 | 0 | Actin [Lepeophtheirus salmonis]. | 1,93 | 34 |
| ACO11823 | 0 | Actin, muscle [Lepeophtheirus salmonis]. | 1,77 | 11 |
| ACO10804 | 4,06617E-59 | Adenosine kinase [Caligus rogercresseyi]. | 1,12 | 1 |
| ACO14812 | 3,1463E-107 | Adenylate kinase isoenzyme 1 [Caligus clemensi]. | 1,47 | 1 |
| ACO14661 | 0 | Alkaline phosphatase, tissue-nonspecific isozyme [Caligus clemensi]. | 1,17 | 1 |
| ACO10437 | 0 | Alpha-amylase A precursor [Caligus rogercresseyi]. | 1,10 | 1 |
| ACO11785 | 1,4109E-120 | Arginine kinase [Lepeophtheirus salmonis]. | 1,92 | 19 |
| ADD38111 | 8,44641E-50 | Beta-crystallin A1 [Lepeophtheirus salmonis]. | 1,19 | 3 |
| AEK11985 | 8,89906E-69 | Calmin-like protein, partial [Tigriopus californicus]. | 2,26 | 7 |
| ACO12974 | 2,6551E-24 | Calreticulin precursor [Lepeophtheirus salmonis]. | 1,16 | 2 |
| ACO10992 | 9,4282E-96 | Carbonic anhydrase 6 [Caligus rogercresseyi]. | 2,53 | 6 |
| ACO15573 | 4,1082E-110 | Catalase [Caligus clemensi]. | 1,36 | 1 |
| ACO11618 | 0 | Cathepsin B precursor [Caligus rogercresseyi]. | 1,05 | 1 |
| ACO15540 | 2,1155E-178 | Cathepsin D precursor [Caligus clemensi]. | 1,06 | 1 |
| ACO10357 | 2,3542E-144 | Cathepsin L precursor [Caligus rogercresseyi]. | 1,34 | 1 |
| ADD24462 | 1,47336E-13 | Cerebellin-3 [Lepeophtheirus salmonis]. | 1,84 | 1 |
| ABU41067 | 2,3212E-177 | Enolase [Lepeophtheirus salmonis]. | 1,11 | 7 |
| ADD38289 | 3,31665E-56 | Gamma-crystallin A [Lepeophtheirus salmonis]. | 1,42 | 4 |
| ABU41084 | 0 | Hypothetical protein [Lepeophtheirus salmonis]. | 1,84 | 15 |
| ACO11368 | 1,5393E-149 | Insulin-like growth factor-binding protein 7 [Caligus rogercresseyi]. | 1,20 | 1 |
| ACO11748 | 0 | Integral membrane protein 2B [Caligus rogercresseyi]. | 1,06 | 1 |
| ACO10640 | 9,5692E-150 | Lethal2essential for life [Caligus rogercresseyi]. | 1,32 | 2 |
| ABU41053 | 8,17972E-38 | Metalloproteinase [Lepeophtheirus salmonis]. | 1,77 | 22 |
| EFX87105 | 0 | Myosin heavy chain isoform 2 [Daphnia pulex]. | 2,02 | 5 |
| ACO12924 | 5,96785E-84 | Myosin light chain alkali [Lepeophtheirus salmonis]. | 1,77 | 15 |
| ACO11734 | 1,7186E-136 | Neuronal calcium sensor 2 [Caligus rogercresseyi]. | 1,55 | 1 |
| ACO11393 | 8,5824E-144 | Neutral sphingomyelinase [Caligus rogercresseyi]. | 1,11 | 1 |
| ACO10282 | 0 | Palmitoyl-protein thioesterase 1 precursor [Caligus rogercresseyi]. | 1,03 | 1 |
| ACO14750 | 2,41E-139 | PDZ and LIM domain protein 1 [Caligus clemensi]. | 1,57 | 1 |
| ACO10787 | 1,8373E-105 | Peflin [Caligus rogercresseyi]. | 1,12 | 1 |
| ADD24486 | 1,3028E-144 | Peritrophin-1 [Lepeophtheirus salmonis]. | 1,16 | 1 |
| BAC66140 | 0 | projectin [Procambarus clarkii]. | 1,95 | 4 |
| ACO10662 | 0 | Tropomodulin [Caligus rogercresseyi]. | 1,77 | 1 |
| ACO14882 | 1,97168E-46 | Tropomyosin [Caligus clemensi]. | 1,55 | 12 |
| ACO15082 | 6,05921E-20 | Troponin C, isoform 1 [Caligus clemensi]. | 1,64 | 5 |
| ACO12887 | 7,21841E-69 | Troponin T [Lepeophtheirus salmonis]. | 1,53 | 11 |
| ABU41102 | 3,04443E-55 | Zeelin1-like protein [Lepeophtheirus salmonis]. | 1,51 | 2 |
| ***Cluster5*** |  |  |  |  |
| ACO11291 | 7,74787E-94 | Cellular retinoic acid-binding protein 2 [Caligus rogercresseyi]. | -1,17 | 15 |
| ABU41064 | 1,2624E-141 | Elongation factor 1-alpha [Lepeophtheirus salmonis]. | -1,24 | 2 |
| AAR01303 | 0 | Elongation factor-2, partial [Mesocyclops edax]. | -1,17 | 1 |
| ACO10379 | 4,529E-93 | Glutathione S-transferase 1-1 [Caligus rogercresseyi]. | -1,14 | 1 |
| ACO12691 | 1,7311E-160 | GTP-binding nuclear protein Ran [Lepeophtheirus salmonis]. | -1,16 | 5 |
| ACO12443 | 6,45367E-59 | Histone H2A [Lepeophtheirus salmonis]. | -1,58 | 2 |
| ACO11965 | 5,3991E-117 | Nascent polypeptide-associated complex [Lepeophtheirus salmonis]. | -1,14 | 4 |
| ACO15498 | 7,033E-142 | Proliferation-associated protein 2G4 [Caligus clemensi]. | -1,32 | 2 |
| ABU41071 | 2,17905E-74 | Receptor for activated protein kinase C-like protein [L. salmonis]. | -1,23 | 2 |
| ACO14986 | 3,34664E-86 | Transcription factor BTF3 homolog 4 [Caligus clemensi]. | -1,13 | 2 |
| ADD24113 | 1,0929E-110 | Translocon-associated protein subunit alpha [L. salmonis]. | -1,16 | 2 |
| ACO10754 | 2,27E-158 | Transport protein Sec61 subunit alpha [Caligus rogercresseyi]. | -1,15 | 5 |
| ACO12709 | 7,14007E-46 | Tubulin alpha-1 chain [Lepeophtheirus salmonis]. | -1,27 | 3 |
| ABU41136 | 0 | vitellogenin-like protein [Lepeophtheirus salmonis]. | -1,32 | 16 |
| ***Cluster6*** |  |  |  |  |
| ACO10287 | 1,02479E-88 | 14-3-3 protein gamma-B [Caligus rogercresseyi]. | 1,37 | 1 |
| ACO11823 | 6,8556E-103 | Actin, muscle [Lepeophtheirus salmonis]. | 1,27 | 1 |
| ABV60390 | 7,0192E-148 | Actin, partial [Artemia franciscana]. | 1,17 | 1 |
| EFX88361 | 2,67283E-33 | Alpha subunit of putative Na+/K+ ATPase [Daphnia pulex]. | 1,14 | 2 |
| ACO13107 | 1,2404E-134 | Antichymotrypsin-2 [Lepeophtheirus salmonis]. | 1,19 | 1 |
| ACO11339 | 1,16864E-68 | B-cell receptor-associated protein 31 [Caligus rogercresseyi]. | 1,06 | 2 |
| ABU41067 | 1,27998E-81 | Enolase [Lepeophtheirus salmonis]. | 1,15 | 4 |
| AGN98124 | 1,64796E-20 | Heat shock protein [Penaeus monodon]. | 1,17 | 2 |
| ABU41053 | 4,56642E-37 | Metalloproteinase [Lepeophtheirus salmonis]. | 1,20 | 6 |
| ACO11221 | 1,774E-27 | Neuronal calcium sensor 2 [Caligus rogercresseyi]. | 1,32 | 2 |
| ABU41130 | 6,77897E-09 | Putative SPT transcription factor family member [L. salmonis]. | 1,21 | 1 |
| ACO11619 | 7,5398E-141 | Sequestosome-1 [Caligus rogercresseyi]. | 1,10 | 3 |
| ACB41380 | 1,32249E-79 | Serine proteinase-like 2b [Pacifastacus leniusculus]. | 1,14 | 2 |
| ACO11350 | 2,16687E-96 | Transaldolase [Caligus rogercresseyi]. | 1,14 | 1 |
