# Supplementary material for: RNA-Seq Analysis Using De Novo Transcriptome Assembly as a Reference for the Salmon Louse Caligus rogercresseyi
Source: PLoS One. 2014 Apr 1;9(4):e92239. doi: 10.1371/journal.pone.0092239 (PMC3972170; doi:10.1371/journal.pone.0092239)
Supplement: File S3 — Table S6. Hypothetical proteins and unannotated contigs Up/down-regulated in Copepodid/Nauplius I–II groups. Table S7. Hypothetical proteins and unannotated contigs Up/down-regulated in Chalimus/Copepodid groups. Table S8. Hypothetical proteins and unannotated contigs Unannotated contigs Up/down-regulated in Male/Female groups. (DOCX) [file pone.0092239.s005.docx]

**Table S6. Hypothetical proteins and unannotated contigs Up/down-regulated in Copepodid/Nauplius I-II groups**

| **Feature ID** | **Lowest E-value** | **LOG2 (fold change)** | **P-value** | **Annotation** |
| --- | --- | --- | --- | --- |
| contig9155 | 0 | 6,1 | 13,7 | hypothetical protein [Lepeophtheirus salmonis]. |
| contig10319 | 0 | 8,4 | 8,6 | not available |
| contig10370 | 0 | 7,9 | 14,7 | not available |
| contig17923 | 0 | 7,3 | 14,6 | not available |
| contig1874 | 0 | 9,0 | 10,6 | not available |
| contig2300 | 0 | 5,1 | 15,4 | not available |
| contig31309 | 0 | 4,1 | 11,7 | not available |
| contig31483 | 0 | 8,4 | 15,7 | not available |
| contig3487 | 0 | 5,1 | 10,5 | not available |
| contig35111 | 0 | 5,3 | 6,9 | not available |
| contig3628 | 0 | 4,4 | 15,4 | not available |
| contig37470 | 0 | 6,8 | 10,3 | not available |
| contig40552 | 0 | 6,6 | 7,2 | not available |
| contig48143 | 0 | 4,1 | 10,3 | not available |
| contig48296 | 0 | 4,4 | 10,6 | not available |
| contig50945 | 0 | 6,1 | 14,8 | not available |
| contig51344 | 0 | 5,5 | 10,7 | not available |
| contig52760 | 0 | 7,4 | 15,7 | not available |
| contig59006 | 0 | 6,2 | 8,6 | not available |
| contig67099 | 0 | 6,3 | 7,5 | not available |
| contig67538 | 0 | 5,5 | 10,7 | not available |
| contig68108 | 0 | 5,8 | 8,2 | not available |
| contig68160 | 0 | 5,5 | 8,4 | not available |
| contig7755 | 0 | 5,5 | 8,2 | not available |
| contig14775 | 2,7753E-149 | 7,4 | 10,4 | hypothetical protein [Lepeophtheirus salmonis]. |
| contig43381 | 3,3029E-143 | 5,0 | 12,6 | hypothetical protein [Lepeophtheirus salmonis]. |
| contig23337 | 1,1263E-102 | 7,9 | 12,6 | hypothetical protein [Lepeophtheirus salmonis]. |
| contig57471 | 8,0239E-93 | 8,2 | 13,5 | hypothetical protein [Lepeophtheirus salmonis]. |
| contig54860 | 1,65195E-84 | 6,7 | 15,1 | hypothetical protein [Lepeophtheirus salmonis]. |
| contig83517 | 2,56684E-78 | 6,5 | 15,0 | hypothetical protein [Lepeophtheirus salmonis]. |
| contig73624 | 7,28474E-77 | 4,9 | 13,3 | hypothetical protein [Lepeophtheirus salmonis]. |
| contig57268 | 8,7956E-74 | 6,5 | 7,5 | hypothetical protein [Lepeophtheirus salmonis]. |
| contig8086 | 3,43975E-68 | 5,0 | 10,2 | hypothetical protein [Lepeophtheirus salmonis]. |
| contig65366 | 2,96132E-64 | 4,4 | 9,9 | hypothetical protein [Lepeophtheirus salmonis]. |
| contig33612 | 3,82811E-54 | 4,1 | 8,2 | hypothetical protein, partial [Lepeophtheirus salmonis]. |
| contig47751 | 1,54973E-48 | 4,8 | 8,2 | hypothetical protein [Lepeophtheirus salmonis]. |
| contig65267 | 6,38195E-46 | 9,1 | 10,5 | hypothetical protein [Lepeophtheirus salmonis]. |
| contig46916 | 3,66505E-42 | 6,8 | 11,0 | hypothetical protein [Lepeophtheirus salmonis]. |
| contig64187 | 1,9E-41 | 7,0 | 10,4 | hypothetical protein [Lepeophtheirus salmonis]. |
| contig56488 | 2,65584E-41 | 5,6 | 12,2 | hypothetical protein [Lepeophtheirus salmonis]. |
| contig46410 | 1,09761E-36 | 6,0 | 8,3 | hypothetical protein [Lepeophtheirus salmonis]. |
| contig21875 | 1,75242E-34 | 6,6 | 9,5 | hypothetical protein [Lepeophtheirus salmonis]. |
| contig57434 | 5,47596E-32 | 4,7 | 9,7 | hypothetical protein [Lepeophtheirus salmonis]. |
| contig18053 | 1,00731E-31 | -5,8 | 14,1 | hypothetical protein DAPPUDRAFT_72393, partial [Daphnia pulex]. |
| contig59153 | 4,14342E-30 | 7,9 | 10,2 | hypothetical protein [Lepeophtheirus salmonis]. |
| contig29321 | 1,14077E-28 | 7,6 | 12,8 | hypothetical protein [Lepeophtheirus salmonis]. |
| contig72917 | 1,16337E-28 | 5,2 | 7,5 | hypothetical protein [Lepeophtheirus salmonis]. |
| contig59069 | 1,72524E-28 | 6,4 | 7,4 | hypothetical protein DAPPUDRAFT_62084, partial [Daphnia pulex]. |
| contig4963 | 6,38157E-25 | -6,2 | 13,0 | hypothetical protein DAPPUDRAFT_69133 [Daphnia pulex]. |
| contig46464 | 8,92663E-21 | 6,2 | 7,4 | hypothetical protein [Lepeophtheirus salmonis]. |
| contig42387 | 2,5831E-20 | 6,7 | 12,7 | hypothetical protein [Lepeophtheirus salmonis]. |
| contig67466 | 1,58567E-18 | 8,4 | 9,7 | hypothetical protein [Lepeophtheirus salmonis]. |
| contig33582 | 1,13688E-17 | 8,1 | 10,0 | hypothetical protein [Lepeophtheirus salmonis]. |
| contig55981 | 4,5783E-17 | 7,0 | 7,6 | hypothetical protein [Lepeophtheirus salmonis]. |
| contig21575 | 3,46922E-16 | -5,1 | 13,9 | hypothetical protein DAPPUDRAFT_37179, partial [Daphnia pulex]. |
| contig41378 | 2,59404E-15 | -4,7 | 12,3 | hypothetical protein DAPPUDRAFT_67127, partial [Daphnia pulex]. |
| contig4964 | 2,61006E-14 | -6,3 | 14,9 | hypothetical protein DAPPUDRAFT_70661, partial [Daphnia pulex]. |
| contig36391 | 1,43989E-11 | 8,2 | 11,5 | hypothetical protein DAPPUDRAFT_52062 [Daphnia pulex]. |
| contig72496 | 7,1488E-10 | 4,3 | 13,7 | hypothetical protein [Lepeophtheirus salmonis]. |
| contig28805 | 4,14667E-09 | 6,9 | 14,1 | hypothetical protein DAPPUDRAFT_301812 [Daphnia pulex]. |
| contig56228 | 7,10061E-07 | 4,0 | 11,6 | hypothetical protein [Lepeophtheirus salmonis]. |
| contig31412 | 2,70515E-06 | 6,4 | 7,0 | hypothetical protein DAPPUDRAFT_301584 [Daphnia pulex]. |

**Table S7. Hypothetical proteins and unannotated contigs Up/down-regulated in Chalimus/Copepodid groups**

| **Feature ID** | **Lowest E-value** | **LOG2 (fold change)** | **P-value** | **Annotation** |
| --- | --- | --- | --- | --- |
| contig43381 | 3,3029E-143 | -6,2 | 6,66134E-16 | hypothetical protein [Lepeophtheirus salmonis]. |
| contig57352 | 2,3351E-81 | -5,3 | 1,10772E-07 | hypothetical protein [Lepeophtheirus salmonis]. |
| contig16144 | 4,05411E-78 | -5,0 | 2,20934E-14 | hypothetical protein [Lepeophtheirus salmonis]. |
| contig25978 | 2,08088E-64 | -7,0 | 7,02993E-13 | hypothetical protein [Lepeophtheirus salmonis]. |
| contig16139 | 7,63772E-52 | -4,6 | 1,04973E-08 | hypothetical protein [Lepeophtheirus salmonis]. |
| contig65267 | 6,38195E-46 | -9,0 | 1,03606E-12 | hypothetical protein [Lepeophtheirus salmonis]. |
| contig46734 | 2,21072E-42 | -8,7 | 1,51E-10 | hypothetical protein [Lepeophtheirus salmonis]. |
| contig8142 | 7,3776E-36 | 6,2 | 0 | hypothetical protein [Lepeophtheirus salmonis]. |
| contig21875 | 1,75242E-34 | -5,1 | 5,34517E-11 | hypothetical protein [Lepeophtheirus salmonis]. |
| contig64534 | 8,57306E-21 | -5,7 | 3,10862E-15 | hypothetical protein [Lepeophtheirus salmonis]. |
| contig27695 | 1,72543E-17 | -6,0 | 3,68872E-12 | hypothetical protein [Lepeophtheirus salmonis]. |
| contig55981 | 4,5783E-17 | -7,5 | 2,26374E-09 | hypothetical protein [Lepeophtheirus salmonis]. |
| contig38419 | 4,88506E-17 | -5,1 | 4,88498E-12 | hypothetical protein [Lepeophtheirus salmonis]. |
| contig4273 | 2,24924E-14 | 6,7 | 1,05094E-12 | hypothetical protein [Lepeophtheirus salmonis]. |
| contig25230 | 6,92689E-08 | -6,8 | 8,52775E-09 | hypothetical protein [Lepeophtheirus salmonis]. |
| contig25902 | 8,60012E-14 | -6,7 | 4,01697E-08 | hypothetical protein DAPPUDRAFT_112150 [Daphnia pulex]. |
| contig19042 | 6,01265E-06 | -8,0 | 1,84464E-12 | hypothetical protein DAPPUDRAFT_112303 [Daphnia pulex]. |
| contig32440 | 3,64986E-82 | -7,7 | 1,09204E-10 | hypothetical protein DAPPUDRAFT_11317 [Daphnia pulex]. |
| contig39695 | 0 | -7,9 | 9,52161E-12 | hypothetical protein DAPPUDRAFT_127538 [Daphnia pulex]. |
| contig17541 | 1,96759E-85 | -5,1 | 1,73857E-10 | hypothetical protein DAPPUDRAFT_203579 [Daphnia pulex]. |
| contig20251 | 0 | -7,4 | 9,99201E-16 | hypothetical protein DAPPUDRAFT_214208 [Daphnia pulex]. |
| contig24327 | 2,6934E-155 | -7,6 | 7,01649E-10 | hypothetical protein DAPPUDRAFT_214360 [Daphnia pulex]. |
| contig31644 | 5,85736E-07 | -8,4 | 6,97693E-09 | hypothetical protein DAPPUDRAFT_214360 [Daphnia pulex]. |
| contig6895 | 6,86736E-78 | 8,2 | 8,21565E-15 | hypothetical protein DAPPUDRAFT_21975 [Daphnia pulex]. |
| contig7276 | 1,60097E-09 | -6,5 | 1,45439E-14 | hypothetical protein DAPPUDRAFT_224358 [Daphnia pulex]. |
| contig2835 | 1,351E-146 | -8,1 | 4,19553E-13 | hypothetical protein DAPPUDRAFT_230054 [Daphnia pulex]. |
| contig14736 | 1,5385E-18 | -4,6 | 1,3343E-11 | hypothetical protein DAPPUDRAFT_230054 [Daphnia pulex]. |
| contig5667 | 9,45449E-18 | -6,0 | 5,73518E-08 | hypothetical protein DAPPUDRAFT_243267 [Daphnia pulex]. |
| contig24919 | 4,04381E-09 | -7,2 | 8,06022E-14 | hypothetical protein DAPPUDRAFT_244593 [Daphnia pulex]. |
| contig13069 | 6,87868E-92 | -4,9 | 2,68674E-14 | hypothetical protein DAPPUDRAFT_245213 [Daphnia pulex]. |
| contig26986 | 0 | -6,5 | 5,2335E-09 | hypothetical protein DAPPUDRAFT_255143 [Daphnia pulex]. |
| contig13298 | 1,3594E-160 | -4,3 | 2,94731E-12 | hypothetical protein DAPPUDRAFT_299840 [Daphnia pulex]. |
| contig7447 | 2,15598E-31 | 5,3 | 1,92335E-09 | hypothetical protein DAPPUDRAFT_301970 [Daphnia pulex]. |
| contig18790 | 7,05634E-38 | -7,0 | 5,34262E-10 | hypothetical protein DAPPUDRAFT_302580 [Daphnia pulex]. |
| contig1147 | 1,31192E-09 | 5,3 | 8,06555E-12 | hypothetical protein DAPPUDRAFT_303213 [Daphnia pulex]. |
| contig47331 | 3,07899E-24 | 6,9 | 1,28586E-11 | hypothetical protein DAPPUDRAFT_304363 [Daphnia pulex]. |
| contig17834 | 0 | -4,3 | 1,39963E-08 | hypothetical protein DAPPUDRAFT_304927 [Daphnia pulex]. |
| contig36184 | 1,7291E-121 | -6,3 | 1,10689E-09 | hypothetical protein DAPPUDRAFT_305317 [Daphnia pulex]. |
| contig2524 | 2,8813E-103 | -4,2 | 6,72569E-09 | hypothetical protein DAPPUDRAFT_305379 [Daphnia pulex]. |
| contig28886 | 1,81292E-69 | -6,2 | 1,69164E-07 | hypothetical protein DAPPUDRAFT_306713 [Daphnia pulex]. |
| contig16948 | 2,13464E-91 | -8,5 | 1,26711E-09 | hypothetical protein DAPPUDRAFT_306978 [Daphnia pulex]. |
| contig10695 | 0 | -5,2 | 2,99757E-10 | hypothetical protein DAPPUDRAFT_310221 [Daphnia pulex]. |
| contig11562 | 7,87991E-54 | -5,2 | 2,44249E-14 | hypothetical protein DAPPUDRAFT_311139 [Daphnia pulex]. |
| contig11563 | 1,40174E-49 | -5,1 | 4,76286E-14 | hypothetical protein DAPPUDRAFT_311139 [Daphnia pulex]. |
| contig15381 | 9,1578E-19 | -4,4 | 2,08589E-12 | hypothetical protein DAPPUDRAFT_314628 [Daphnia pulex]. |
| contig24694 | 2,70669E-20 | -8,1 | 1,70863E-13 | hypothetical protein DAPPUDRAFT_315544 [Daphnia pulex]. |
| contig7467 | 3,84442E-06 | -5,8 | 1,4766E-14 | hypothetical protein DAPPUDRAFT_317149 [Daphnia pulex]. |
| contig2329 | 9,4664E-92 | 8,3 | 3,10862E-15 | hypothetical protein DAPPUDRAFT_318553 [Daphnia pulex]. |
| contig12580 | 4,18518E-67 | -5,0 | 1,38511E-10 | hypothetical protein DAPPUDRAFT_320238 [Daphnia pulex]. |
| contig32208 | 1,015E-137 | -5,1 | 4,83012E-10 | hypothetical protein DAPPUDRAFT_321683 [Daphnia pulex]. |
| contig35117 | 1,47482E-36 | -5,4 | 7,10543E-15 | hypothetical protein DAPPUDRAFT_321683 [Daphnia pulex]. |
| contig22592 | 3,37451E-08 | -6,5 | 9,54792E-15 | hypothetical protein DAPPUDRAFT_323345 [Daphnia pulex]. |
| contig4840 | 1,73858E-74 | 8,9 | 7,8253E-12 | hypothetical protein DAPPUDRAFT_326182 [Daphnia pulex]. |
| contig9263 | 5,95688E-65 | 6,8 | 2,2085E-11 | hypothetical protein DAPPUDRAFT_326182 [Daphnia pulex]. |
| contig1186 | 2,7885E-146 | 8,5 | 2,15565E-09 | hypothetical protein DAPPUDRAFT_327607 [Daphnia pulex]. |
| contig38524 | 3,13565E-05 | -7,0 | 8,17651E-10 | hypothetical protein DAPPUDRAFT_333111 [Daphnia pulex]. |
| contig15598 | 1,9418E-104 | -6,3 | 1,04954E-09 | hypothetical protein DAPPUDRAFT_45422 [Daphnia pulex]. |
| contig33865 | 3,72535E-43 | -6,2 | 3,92775E-12 | hypothetical protein DAPPUDRAFT_47227 [Daphnia pulex]. |
| contig18701 | 9,7121E-137 | -6,3 | 3,82174E-10 | hypothetical protein DAPPUDRAFT_49881[Daphnia pulex]. |
| contig29286 | 4,9988E-126 | -7,6 | 8,10463E-14 | hypothetical protein DAPPUDRAFT_50825 [Daphnia pulex]. |
| contig1420 | 9,62726E-31 | 9,3 | 4,44089E-15 | hypothetical protein DAPPUDRAFT_51887 [Daphnia pulex]. |
| contig11245 | 1,52684E-93 | -4,8 | 3,20854E-14 | hypothetical protein DAPPUDRAFT_53984 [Daphnia pulex]. |
| contig11242 | 2,69869E-89 | -5,3 | 3,66374E-15 | hypothetical protein DAPPUDRAFT_53984 [Daphnia pulex]. |
| contig595 | 4,093E-137 | -6,5 | 7,36938E-11 | hypothetical protein DAPPUDRAFT_54063 [Daphnia pulex]. |
| contig17286 | 1,48E-128 | -5,4 | 3,59664E-09 | hypothetical protein DAPPUDRAFT_54063 [Daphnia pulex]. |
| contig33546 | 6,65196E-39 | -8,5 | 1,20141E-09 | hypothetical protein DAPPUDRAFT_56607 [Daphnia pulex]. |
| contig59069 | 1,72524E-28 | -6,9 | 3,26499E-09 | hypothetical protein DAPPUDRAFT_62084 [Daphnia pulex]. |
| contig10503 | 1,38044E-57 | -6,4 | 2,36923E-10 | hypothetical protein DAPPUDRAFT_93942 [Daphnia pulex]. |
| contig35394 | 1,30351E-21 | -9,3 | 3,44169E-15 | hypothetical protein DAPPUDRAFT_98236 [Daphnia pulex]. |
| contig32636 | 2,47781E-20 | -8,5 | 2,53265E-09 | hypothetical protein DAPPUDRAFT_98236 [Daphnia pulex]. |
| contig19815 | 1,27539E-40 | -5,1 | 1,21455E-07 | hypothetical protein DAPPUDRAFT_99801 [Daphnia pulex]. |
| contig41889 | 1,44627E-23 | -8,6 | 9,20644E-10 | hypothetical protein, partial [Lepeophtheirus salmonis]. |
| contig42473 | 1,37131E-14 | -6,7 | 3,07417E-08 | hypothetical protein, partial [Lepeophtheirus salmonis]. |
| contig4444 | 0 | 7,9 | 0 | not available |
| contig29674 | 0 | 9,5 | 0 | not available |
| contig31352 | 0 | -7,4 | 5,55112E-16 | not available |
| contig13848 | 0 | -6,0 | 6,66134E-16 | not available |
| contig56998 | 0 | -4,2 | 6,66134E-16 | not available |
| contig9625 | 0 | 7,0 | 1,77636E-15 | not available |
| contig42353 | 0 | 9,3 | 2,66454E-15 | not available |
| contig23217 | 0 | -8,3 | 5,55112E-15 | not available |
| contig40449 | 0 | -7,7 | 1,03251E-14 | not available |
| contig22547 | 0 | 6,3 | 1,42109E-14 | not available |
| contig48143 | 0 | -9,2 | 2,07612E-14 | not available |
| contig42523 | 0 | 8,2 | 4,77396E-14 | not available |
| contig8457 | 0 | -5,7 | 8,40439E-14 | not available |
| contig6611 | 0 | 9,1 | 9,92539E-14 | not available |
| contig7552 | 0 | -5,7 | 3,49165E-13 | not available |
| contig14783 | 0 | -4,7 | 9,4269E-13 | not available |
| contig19615 | 0 | 8,0 | 1,4766E-12 | not available |
| contig33252 | 0 | -6,7 | 1,54243E-12 | not available |
| contig1296 | 0 | 7,0 | 1,71596E-12 | not available |
| contig43502 | 0 | -4,7 | 2,18692E-12 | not available |
| contig12240 | 0 | -7,9 | 3,30169E-12 | not available |
| contig15302 | 0 | -4,4 | 4,83003E-12 | not available |
| contig12363 | 0 | 6,3 | 6,45972E-12 | not available |
| contig33610 | 0 | -6,1 | 7,02971E-12 | not available |
| contig13861 | 0 | -5,3 | 8,52785E-12 | not available |
| contig13856 | 0 | -5,9 | 1,00799E-11 | not available |
| contig289 | 0 | 5,4 | 1,39102E-11 | not available |
| contig6034 | 0 | 8,8 | 4,83003E-11 | not available |
| contig72929 | 0 | -4,5 | 6,8316E-11 | not available |
| contig24178 | 0 | -4,9 | 7,1069E-11 | not available |
| contig68160 | 0 | -8,7 | 7,5716E-11 | not available |
| contig18837 | 0 | -6,4 | 1,18807E-10 | not available |
| contig308 | 0 | -8,7 | 1,22099E-10 | not available |
| contig36650 | 0 | -8,7 | 2,70901E-10 | not available |
| contig18527 | 0 | 8,6 | 3,46757E-10 | not available |
| contig38339 | 0 | -4,3 | 3,84038E-10 | not available |
| contig28254 | 0 | -4,8 | 4,28934E-10 | not available |
| contig41466 | 0 | 8,6 | 5,40619E-10 | not available |
| contig28873 | 0 | -5,2 | 6,05971E-10 | not available |
| contig29016 | 0 | -6,3 | 6,85813E-10 | not available |
| contig61164 | 0 | -4,9 | 7,15058E-10 | not available |
| contig6769 | 0 | 7,0 | 8,05231E-10 | not available |
| contig5022 | 0 | 6,6 | 8,89044E-10 | not available |
| contig64891 | 0 | -4,4 | 9,24437E-10 | not available |
| contig26641 | 0 | -4,6 | 9,31141E-10 | not available |
| contig46605 | 0 | -7,5 | 1,55914E-09 | not available |
| contig4242 | 0 | 5,6 | 1,94209E-09 | not available |
| contig60339 | 0 | -4,7 | 2,3338E-09 | not available |
| contig1310 | 0 | 8,5 | 3,36423E-09 | not available |
| contig36889 | 0 | 7,5 | 3,91076E-09 | not available |
| contig35111 | 0 | -8,4 | 4,3164E-09 | not available |
| contig1309 | 0 | 8,4 | 4,52714E-09 | not available |
| contig24145 | 0 | -4,9 | 6,0705E-09 | not available |
| contig23216 | 0 | -7,4 | 6,23452E-09 | not available |
| contig27503 | 0 | -6,8 | 8,52775E-09 | not available |
| contig35792 | 0 | -7,4 | 9,55541E-09 | not available |
| contig55389 | 0 | -8,3 | 1,55445E-08 | not available |
| contig36177 | 0 | 8,3 | 2,69574E-08 | not available |
| contig55958 | 0 | 8,3 | 2,69574E-08 | not available |
| contig1137 | 0 | 6,7 | 2,70284E-08 | not available |
| contig40751 | 0 | 6,3 | 2,83907E-08 | not available |
| contig32815 | 0 | -8,3 | 3,65756E-08 | not available |
| contig7055 | 0 | -4,8 | 4,66322E-08 | not available |
| contig42300 | 0 | -7,2 | 4,75138E-08 | not available |
| contig24267 | 0 | -8,2 | 4,7801E-08 | not available |
| contig5491 | 0 | -4,3 | 4,84079E-08 | not available |
| contig24177 | 0 | -7,2 | 5,57937E-08 | not available |
| contig11008 | 0 | -4,7 | 6,64769E-08 | not available |
| contig26484 | 0 | -5,9 | 6,73405E-08 | not available |
| contig26701 | 0 | -5,1 | 8,81312E-08 | not available |
| contig26800 | 0 | -4,7 | 8,84033E-08 | not available |
| contig29098 | 0 | -5,2 | 1,30058E-07 | not available |
| contig20947 | 0 | -4,9 | 1,33011E-07 | not available |
| contig83674 | 2,9149E-110 | 5,5 | 3,68594E-14 | unnamed protein product [Lepeophtheirus salmonis]. |
| contig16204 | 8,63797E-85 | 9,5 | 0 | unnamed protein product [Lepeophtheirus salmonis]. |
| contig83425 | 4,8225E-79 | 7,4 | 9,07353E-09 | unnamed protein product [Lepeophtheirus salmonis]. |
| contig20379 | 1,73948E-78 | 5,9 | 1,16076E-11 | unnamed protein product [Lepeophtheirus salmonis]. |
| contig30660 | 1,32473E-75 | 7,4 | 1,05275E-08 | unnamed protein product [Lepeophtheirus salmonis]. |
| contig10163 | 2,99E-72 | 9,0 | 1,40243E-12 | unnamed protein product [Lepeophtheirus salmonis]. |
| contig23061 | 6,49126E-72 | 8,9 | 2,52776E-12 | unnamed protein product [Lepeophtheirus salmonis]. |
| contig23760 | 7,12406E-67 | 7,7 | 1,65886E-10 | unnamed protein product [Lepeophtheirus salmonis]. |
| contig37050 | 4,93077E-66 | 6,7 | 2,70284E-08 | unnamed protein product [Lepeophtheirus salmonis]. |
| contig567 | 1,53383E-61 | 8,4 | 4,44089E-16 | unnamed protein product [Lepeophtheirus salmonis]. |
| contig33493 | 1,20801E-56 | 8,3 | 8,88178E-16 | unnamed protein product [Lepeophtheirus salmonis]. |
| contig25875 | 7,09593E-54 | 6,4 | 1,90928E-08 | unnamed protein product [Lepeophtheirus salmonis]. |
| contig30955 | 4,82125E-52 | 7,6 | 7,65486E-10 | unnamed protein product [Lepeophtheirus salmonis]. |
| contig40860 | 5,39007E-52 | 8,4 | 1,05064E-08 | unnamed protein product [Lepeophtheirus salmonis]. |
| contig28004 | 1,43547E-47 | 7,8 | 2,55354E-11 | unnamed protein product [Lepeophtheirus salmonis]. |
| contig21489 | 1,52116E-38 | 8,7 | 2,01572E-10 | unnamed protein product [Lepeophtheirus salmonis]. |
| contig44771 | 1,29275E-27 | 8,1 | 2,40252E-13 | unnamed protein product [Lepeophtheirus salmonis]. |
| contig12368 | 4,83469E-25 | 9,4 | 0 | unnamed protein product [Lepeophtheirus salmonis]. |
| contig30982 | 2,77502E-17 | 6,5 | 5,01335E-09 | unnamed protein product [Lepeophtheirus salmonis]. |
| contig17457 | 2,2919E-15 | 7,3 | 2,66454E-15 | unnamed protein product [Lepeophtheirus salmonis]. |

**Table S8. Hypothetical proteins and unannotated contigs Unannotated contigs Up/down-regulated in Male/Female groups**

| **Feature ID** | **Lowest E-value** | **LOG2 (fold change)** | **P-value** | **Annotation** |
| --- | --- | --- | --- | --- |
| contig23911 | 9,01787E-86 | 4,5 | 7,77156E-15 | hypothetical protein [Lepeophtheirus salmonis]. |
| contig21593 | 3,1039E-103 | 4,6 | 8,02571E-09 | hypothetical protein [Lepeophtheirus salmonis]. |
| contig36024 | 7,29868E-18 | 5,2 | 1,39888E-14 | hypothetical protein [Lepeophtheirus salmonis]. |
| contig57196 | 2,59583E-67 | 5,3 | 1,55431E-15 | hypothetical protein [Lepeophtheirus salmonis]. |
| contig63696 | 4,68886E-43 | 5,7 | 2,54226E-10 | hypothetical protein [Lepeophtheirus salmonis]. |
| contig21736 | 3,65271E-61 | 6,1 | 2,67084E-11 | hypothetical protein [Lepeophtheirus salmonis]. |
| contig61342 | 0,00283928 | 6,2 | 1,74476E-09 | hypothetical protein [Lepeophtheirus salmonis]. |
| contig47896 | 5,85139E-37 | 6,2 | 2,15383E-14 | hypothetical protein [Lepeophtheirus salmonis]. |
| contig56604 | 1,07854E-08 | 7,4 | 1,13567E-08 | hypothetical protein [Lepeophtheirus salmonis]. |
| contig48455 | 0,00116034 | 7,8 | 2,22422E-11 | hypothetical protein [Lepeophtheirus salmonis]. |
| contig56228 | 7,10061E-07 | 8,5 | 3,46649E-09 | hypothetical protein [Lepeophtheirus salmonis]. |
| contig53343 | 3,0154E-86 | 9,0 | 1,32583E-12 | hypothetical protein [Lepeophtheirus salmonis]. |
| contig506 | 3,07601E-07 | -4,7 | 7,21645E-15 | hypothetical protein DAPPUDRAFT_113123 [Daphnia pulex]. |
| contig4940 | 1,10177E-28 | -7,2 | 4,71228E-08 | hypothetical protein DAPPUDRAFT_12194 [Daphnia pulex]. |
| contig4852 | 0 | -8,4 | 7,47042E-09 | hypothetical protein DAPPUDRAFT_187662 [Daphnia pulex]. |
| contig53845 | 6,51673E-10 | 4,8 | 5,17072E-08 | hypothetical protein DAPPUDRAFT_209558 [Daphnia pulex]. |
| contig41471 | 7,71176E-10 | 4,8 | 8,67866E-11 | hypothetical protein DAPPUDRAFT_209558 [Daphnia pulex]. |
| contig7270 | 1,17151E-15 | -7,8 | 6,52388E-11 | hypothetical protein DAPPUDRAFT_213023 [Daphnia pulex]. |
| contig7410 | 6,37547E-31 | -5,5 | 4,89608E-14 | hypothetical protein DAPPUDRAFT_23978 [Daphnia pulex]. |
| contig8367 | 0,513197 | -4,6 | 2,63678E-13 | hypothetical protein DAPPUDRAFT_308213 [Daphnia pulex]. |
| contig24247 | 3,62794E-15 | 8,3 | 2,66454E-15 | hypothetical protein DAPPUDRAFT_309362 [Daphnia pulex]. |
| contig8499 | 1,4767 | -5,6 | 2,69607E-12 | hypothetical protein DAPPUDRAFT_309815 [Daphnia pulex]. |
| contig59591 | 8,03988 | 8,0 | 8,78408E-13 | hypothetical protein DAPPUDRAFT_313245 [Daphnia pulex]. |
| contig3419 | 5,08024 | -9,1 | 1,09801E-13 | hypothetical protein DAPPUDRAFT_313644 [Daphnia pulex]. |
| contig46171 | 0,00176359 | 6,9 | 2,66239E-09 | hypothetical protein DAPPUDRAFT_316923 [Daphnia pulex]. |
| contig46170 | 0,000650108 | 8,5 | 1,11359E-09 | hypothetical protein DAPPUDRAFT_316923 [Daphnia pulex]. |
| contig4526 | 1,9526E-105 | -7,6 | 1,77636E-14 | hypothetical protein DAPPUDRAFT_317027 [Daphnia pulex]. |
| contig7944 | 3,23844E-97 | -6,3 | 2,28533E-08 | hypothetical protein DAPPUDRAFT_319730 [Daphnia pulex]. |
| contig3827 | 3,40176E-26 | -9,4 | 1,11022E-16 | hypothetical protein DAPPUDRAFT_323318 [Daphnia pulex]. |
| contig2281 | 5,108E-117 | -8,2 | 4,25897E-08 | hypothetical protein DAPPUDRAFT_330715 [Daphnia pulex]. |
| contig2282 | 9,3176E-121 | -6,8 | 9,13286E-09 | hypothetical protein DAPPUDRAFT_330715 [Daphnia pulex]. |
| contig4603 | 1,85076E-44 | 5,7 | 7,11327E-10 | hypothetical protein DAPPUDRAFT_43160 [Daphnia pulex]. |
| contig41106 | 3,77939E-40 | 7,3 | 5,32907E-15 | hypothetical protein DAPPUDRAFT_58486 [Daphnia pulex]. |
| contig41936 | 2,01099E-10 | -6,1 | 9,53455E-09 | hypothetical protein DAPPUDRAFT_96143 [Daphnia pulex]. |
| contig52519 | 8,73864E-19 | 5,9 | 5,39451E-09 | hypothetical protein, partial [Lepeophtheirus salmonis]. |
| contig40898 | 0,0230551 | 5,9 | 3,95763E-09 | hypothetical protein, partial [Lepeophtheirus salmonis]. |
| contig1539 | 0 | -9,4 | 2,22045E-16 | not available |
| contig2427 | 0 | -9,4 | 4,44089E-16 | not available |
| contig2955 | 0 | -9,0 | 5,01155E-13 | not available |
| contig3014 | 0 | -9,0 | 1,31195E-12 | not available |
| contig1144 | 0 | -8,7 | 9,80622E-11 | not available |
| contig5072 | 0 | -8,4 | 1,11022E-16 | not available |
| contig9999 | 0 | -8,2 | 5,5039E-08 | not available |
| contig26236 | 0 | -8,0 | 5,26912E-13 | not available |
| contig9654 | 0 | -7,8 | 4,34457E-11 | not available |
| contig2776 | 0 | -7,5 | 1,0425E-13 | not available |
| contig1310 | 0 | -7,3 | 3,55271E-15 | not available |
| contig1137 | 0 | -7,3 | 4,10783E-15 | not available |
| contig2833 | 0 | -7,0 | 5,244E-10 | not available |
| contig3739 | 0 | -7,0 | 5,51802E-10 | not available |
| contig6778 | 0 | -6,7 | 3,4794E-10 | not available |
| contig30169 | 0 | -6,3 | 6,38518E-10 | not available |
| contig1495 | 0 | -6,1 | 8,99281E-15 | not available |
| contig2273 | 0 | -5,8 | 2,6501E-13 | not available |
| contig6769 | 0 | -5,0 | 6,08513E-12 | not available |
| contig8808 | 0 | -5,0 | 6,13509E-13 | not available |
| contig5486 | 0 | -4,8 | 1,25051E-10 | not available |
| contig1648 | 0 | -4,5 | 4,4038E-08 | not available |
| contig39842 | 0 | 4,5 | 6,19751E-10 | not available |
| contig10875 | 0 | 4,6 | 2,07946E-09 | not available |
| contig42868 | 0 | 4,9 | 6,26166E-13 | not available |
| contig16897 | 0 | 5,0 | 1,33227E-15 | not available |
| contig56517 | 0 | 5,0 | 7,54574E-12 | not available |
| contig36666 | 0 | 5,0 | 3,80584E-13 | not available |
| contig34742 | 0 | 5,0 | 5,01243E-12 | not available |
| contig30406 | 0 | 5,4 | 3,57974E-10 | not available |
| contig12707 | 0 | 5,6 | 1,67124E-10 | not available |
| contig20383 | 0 | 5,7 | 2,45137E-13 | not available |
| contig56998 | 0 | 5,8 | 2,22045E-16 | not available |
| contig50595 | 0 | 5,9 | 3,95763E-09 | not available |
| contig54606 | 0 | 6,1 | 5,12923E-13 | not available |
| contig57095 | 0 | 6,1 | 3,89214E-10 | not available |
| contig83323 | 0 | 6,2 | 4,65122E-09 | not available |
| contig30059 | 0 | 6,6 | 4,83517E-10 | not available |
| contig58922 | 0 | 7,2 | 2,30926E-14 | not available |
| contig15282 | 0 | 7,3 | 2,34441E-08 | not available |
| contig36679 | 0 | 7,6 | 1,05527E-09 | not available |
| contig56909 | 0 | 7,6 | 4,39362E-10 | not available |
| contig308 | 0 | 7,8 | 1,81117E-11 | not available |
| contig42318 | 0 | 8,3 | 1,33227E-15 | not available |
| contig32973 | 0 | 8,5 | 1,44127E-09 | not available |
| contig41447 | 0 | 8,8 | 2,73692E-11 | not available |
| contig63650 | 0 | 8,9 | 6,84208E-12 | not available |
| contig56046 | 0 | 9,1 | 3,49942E-13 | not available |
| contig36177 | 0 | 9,1 | 2,32259E-13 | not available |
| contig22539 | 4,7966E-79 | -9,4 | 1,11022E-16 | unnamed protein product [Lepeophtheirus salmonis]. |
| contig11723 | 1,23385E-42 | -9,4 | 2,22045E-16 | unnamed protein product [Lepeophtheirus salmonis]. |
| contig34572 | 0,0011894 | -9,2 | 1,77636E-14 | unnamed protein product [Lepeophtheirus salmonis]. |
| contig40860 | 5,39007E-52 | -9,1 | 3,02092E-13 | unnamed protein product [Lepeophtheirus salmonis]. |
| contig37056 | 1,90673E-35 | -8,9 | 2,80587E-12 | unnamed protein product [Lepeophtheirus salmonis]. |
| contig31928 | 2,21077E-35 | -8,7 | 2,2131E-10 | unnamed protein product [Lepeophtheirus salmonis]. |
| contig81220 | 2,0379E-70 | -8,5 | 2,55537E-09 | unnamed protein product [Lepeophtheirus salmonis]. |
| contig31234 | 8,68309E-09 | -8,4 | 1,11022E-16 | unnamed protein product [Lepeophtheirus salmonis]. |
| contig304 | 5,21942E-65 | -8,4 | 1,11022E-16 | unnamed protein product [Lepeophtheirus salmonis]. |
| contig14891 | 3,6532E-67 | -8,4 | 4,44089E-16 | unnamed protein product [Lepeophtheirus salmonis]. |
| contig26905 | 9,0645E-58 | -8,3 | 9,99201E-16 | unnamed protein product [Lepeophtheirus salmonis]. |
| contig30955 | 4,82125E-52 | -8,3 | 2,55112E-08 | unnamed protein product [Lepeophtheirus salmonis]. |
| contig83453 | 6,09893E-10 | -8,3 | 3,84391E-08 | unnamed protein product [Lepeophtheirus salmonis]. |
| contig23061 | 6,49126E-72 | -8,2 | 5,5039E-08 | unnamed protein product [Lepeophtheirus salmonis]. |
| contig83893 | 4,36821E-33 | -8,2 | 5,5039E-08 | unnamed protein product [Lepeophtheirus salmonis]. |
| contig27285 | 1,88759E-16 | -8,2 | 4,64073E-14 | unnamed protein product [Lepeophtheirus salmonis]. |
| contig83891 | 1,60046E-65 | -8,0 | 8,31113E-13 | unnamed protein product [Lepeophtheirus salmonis]. |
| contig23186 | 9,7188E-122 | -7,8 | 4,34457E-11 | unnamed protein product [Lepeophtheirus salmonis]. |
| contig16351 | 1,93006E-52 | -7,7 | 2,22045E-15 | unnamed protein product [Lepeophtheirus salmonis]. |
| contig19199 | 2,33808E-20 | -7,6 | 1,18754E-09 | unnamed protein product [Lepeophtheirus salmonis]. |
| contig18986 | 1,2259E-101 | -7,4 | 7,46145E-09 | unnamed protein product [Lepeophtheirus salmonis]. |
| contig16119 | 6,7438E-111 | -7,3 | 2,42051E-08 | unnamed protein product [Lepeophtheirus salmonis]. |
| contig83398 | 1,3858E-105 | -7,3 | 9,45699E-12 | unnamed protein product [Lepeophtheirus salmonis]. |
| contig47393 | 0,000170931 | -7,1 | 4,07674E-13 | unnamed protein product [Lepeophtheirus salmonis]. |
| contig33335 | 8,27862E-43 | -6,9 | 3,78697E-12 | unnamed protein product [Lepeophtheirus salmonis]. |
| contig46177 | 9,65911E-48 | -6,7 | 2,17935E-08 | unnamed protein product [Lepeophtheirus salmonis]. |
| contig39427 | 3,21064E-07 | -6,3 | 1,2701E-11 | unnamed protein product [Lepeophtheirus salmonis]. |
| contig17457 | 2,2919E-15 | -5,8 | 2,37948E-08 | unnamed protein product [Lepeophtheirus salmonis]. |
| contig35692 | 5,06907E-35 | -5,0 | 2,64828E-09 | unnamed protein product [Lepeophtheirus salmonis]. |
